# Supplementary figures and images for: Rubus coreanus extract prevents kidney fibrosis through TGF-β/Smad pathway inhibition
Source: PLoS One. 2025 May 12;20(5):e0321282. doi: 10.1371/journal.pone.0321282 (PMC12068887; doi:10.1371/journal.pone.0321282)

Supplementary Figure 1

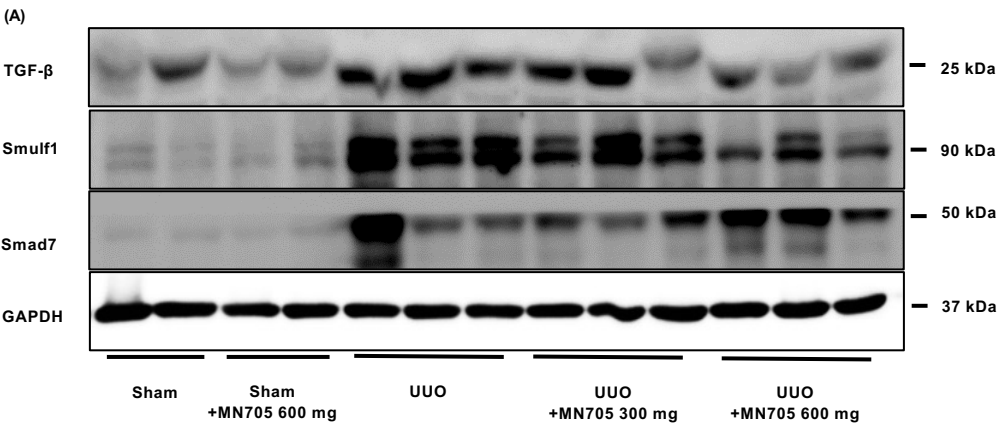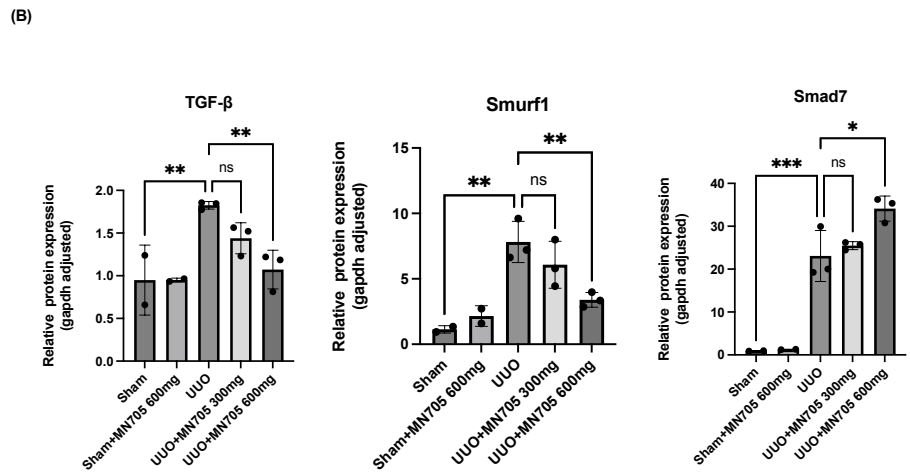

Supplement: S1 Fig — (A) Protein expression of TGF-β, Smurf1, and Smad7. (B) Measurement of western blot results. The data are presented as the mean ± SD of per group. *P < 0.05, ** P < 0.01, ***P < 0.001. (PDF) [file pone.0321282.s001.pdf]
