# Supplementary material for: Rubus coreanus extract prevents kidney fibrosis through TGF-β/Smad pathway inhibition
Source: PLoS One. 2025 May 12;20(5):e0321282. doi: 10.1371/journal.pone.0321282 (PMC12068887; doi:10.1371/journal.pone.0321282)

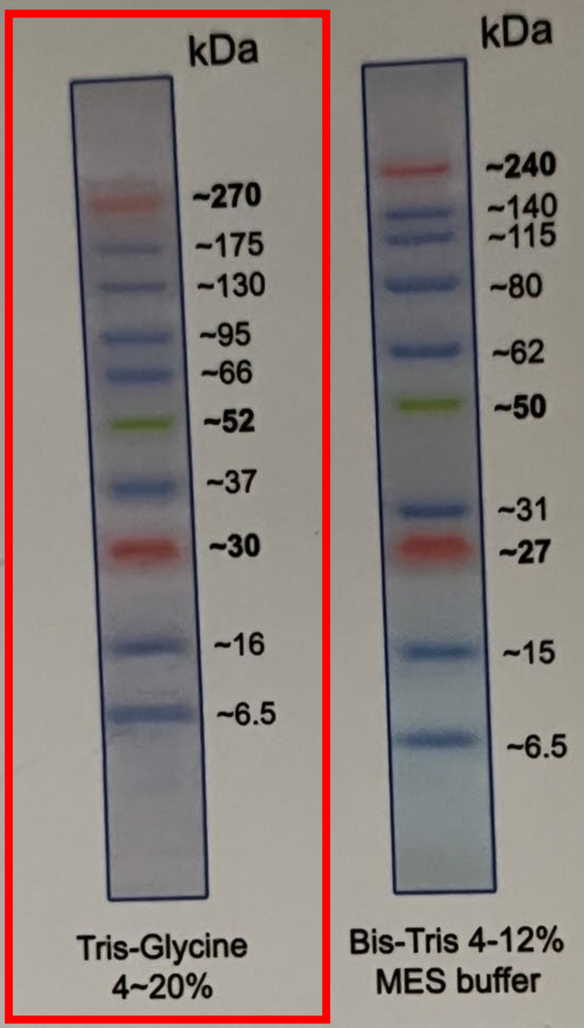

Note. The apparent molecular weight (kDa) of each protein has been determined by calibration against unstained protein standards; supplemental data should be considered for more accurate adjustment in different electrophoresis conditions.

All products are for research use only.

Caution: Not intended for human or animal diagnostic or therapeutic uses.

www.nippongenetics.eu
www.nippongenetics.de

Fig. 2

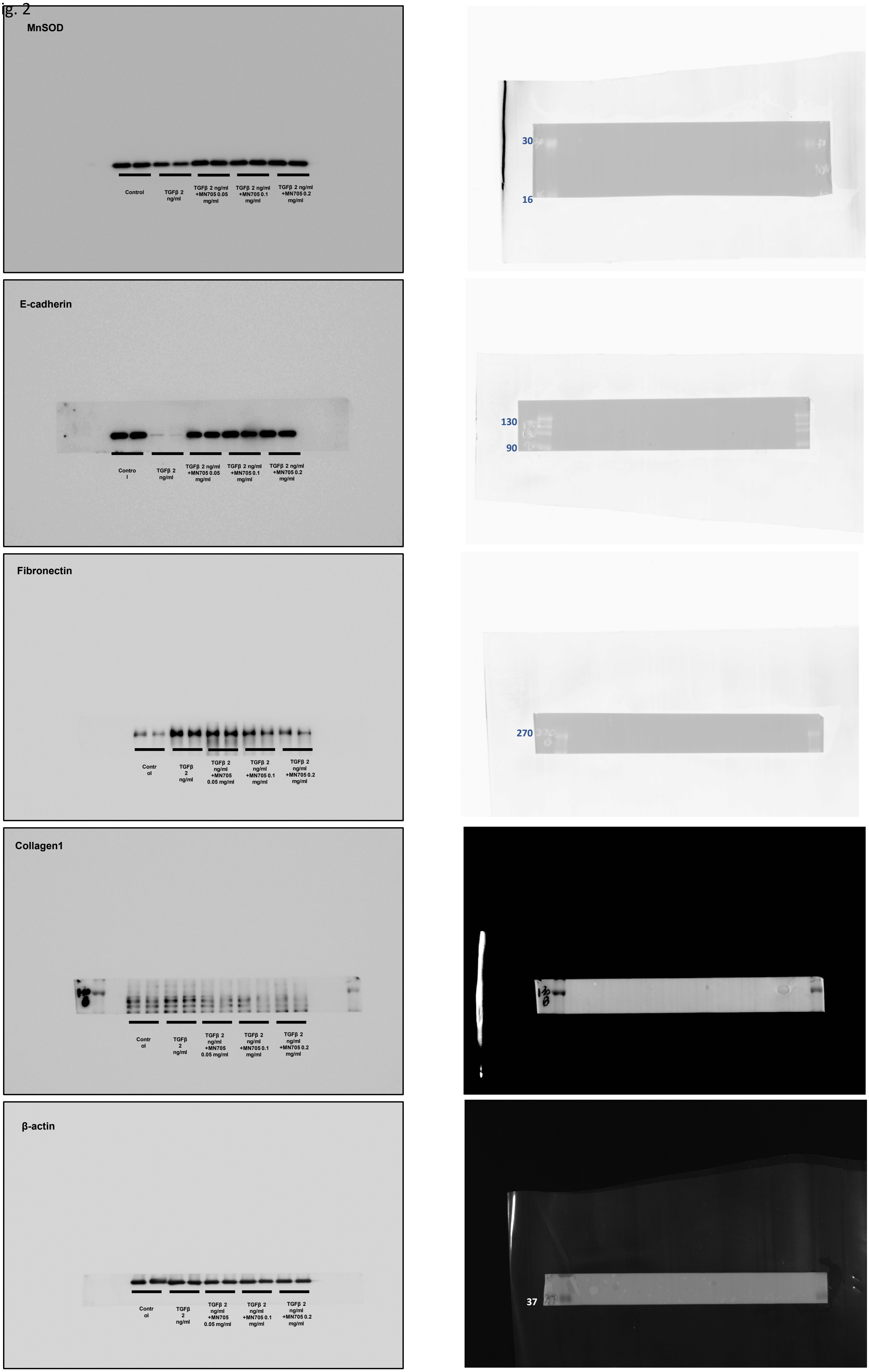
MnSOD
Control
TGFβ 2 ng/ml
TGFβ 2 ng/ml +MN705 0.05 mg/ml
TGFβ 2 ng/ml +MN705 0.1 mg/ml
TGFβ 2 ng/ml +MN705 0.2 mg/ml
30
16
E-cadherin
Contro l
TGFβ 2 ng/ml
TGFβ 2 ng/ml +MN705 0.05 mg/ml
TGFβ 2 ng/ml +MN705 0.1 mg/ml
TGFβ 2 ng/ml +MN705 0.2 mg/ml
130
90
Fibronectin
Contr ol
TGFβ 2 ng/ml
TGFβ 2 ng/ml +MN705 0.05 mg/ml
TGFβ 2 ng/ml +MN705 0.1 mg/ml
TGFβ 2 ng/ml +MN705 0.2 mg/ml
270
Collagen1
Contr ol
TGFβ 2 ng/ml
TGFβ 2 ng/ml +MN705 0.05 mg/ml
TGFβ 2 ng/ml +MN705 0.1 mg/ml
TGFβ 2 ng/ml +MN705 0.2 mg/ml
β-actin
Contr ol
TGFβ 2 ng/ml
TGFβ 2 ng/ml +MN705 0.05 mg/ml
TGFβ 2 ng/ml +MN705 0.1 mg/ml
TGFβ 2 ng/ml +MN705 0.2 mg/ml
37

Fig. 2(Only for statistics not used in main figure)

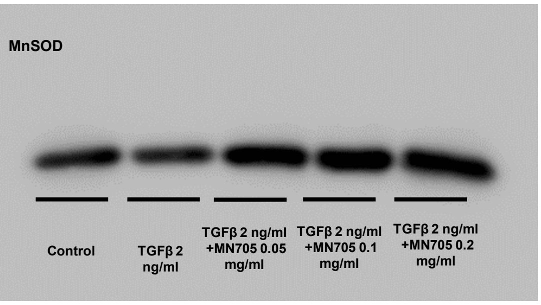

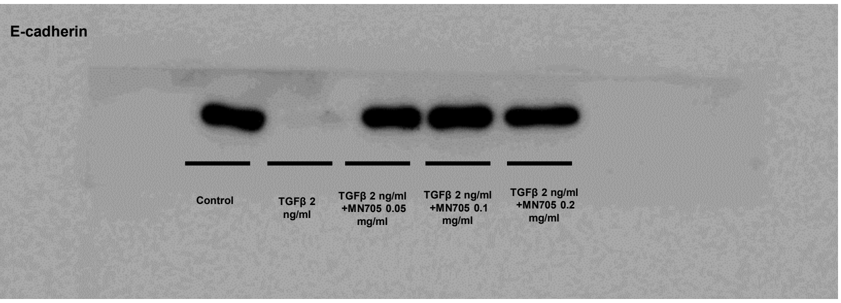

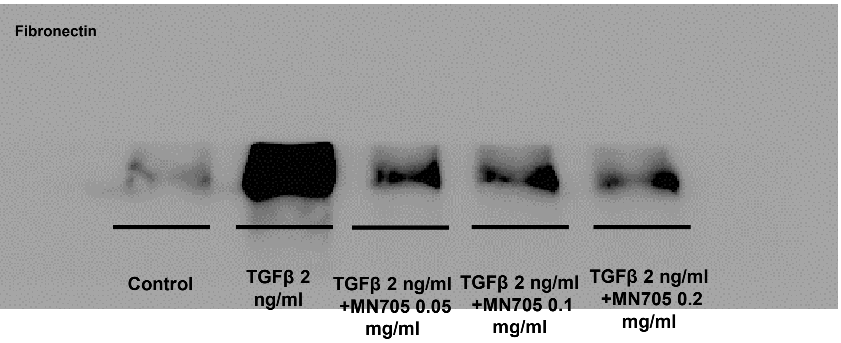

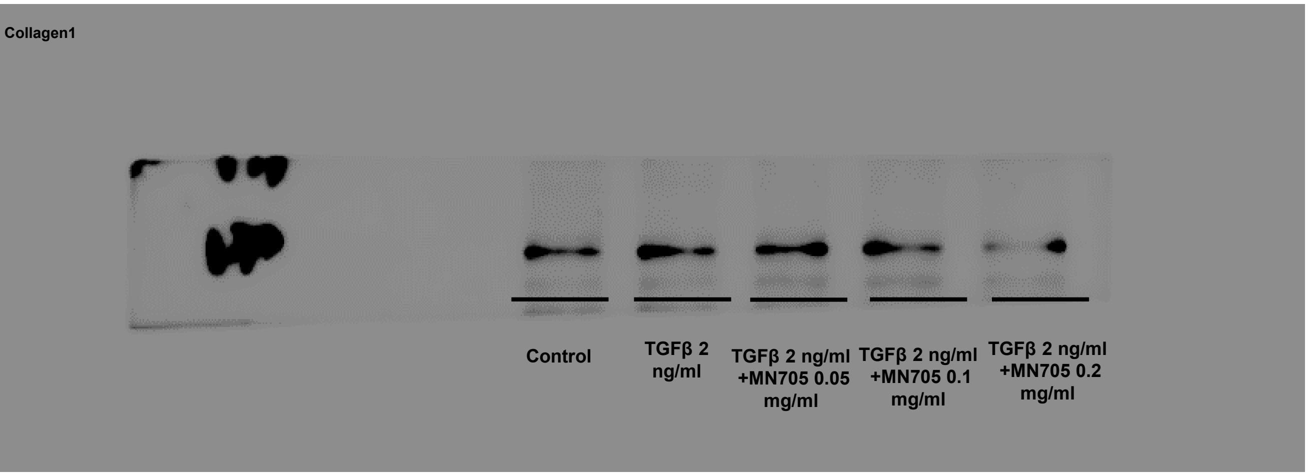

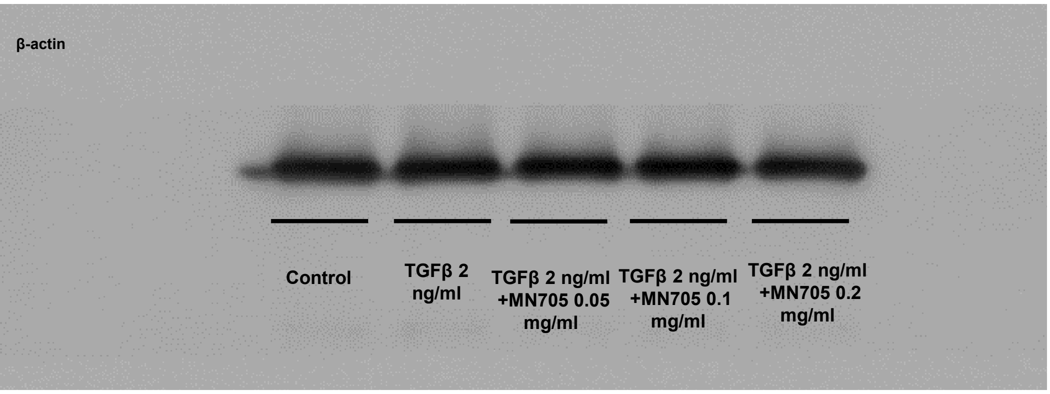

Fig. 3 A/B

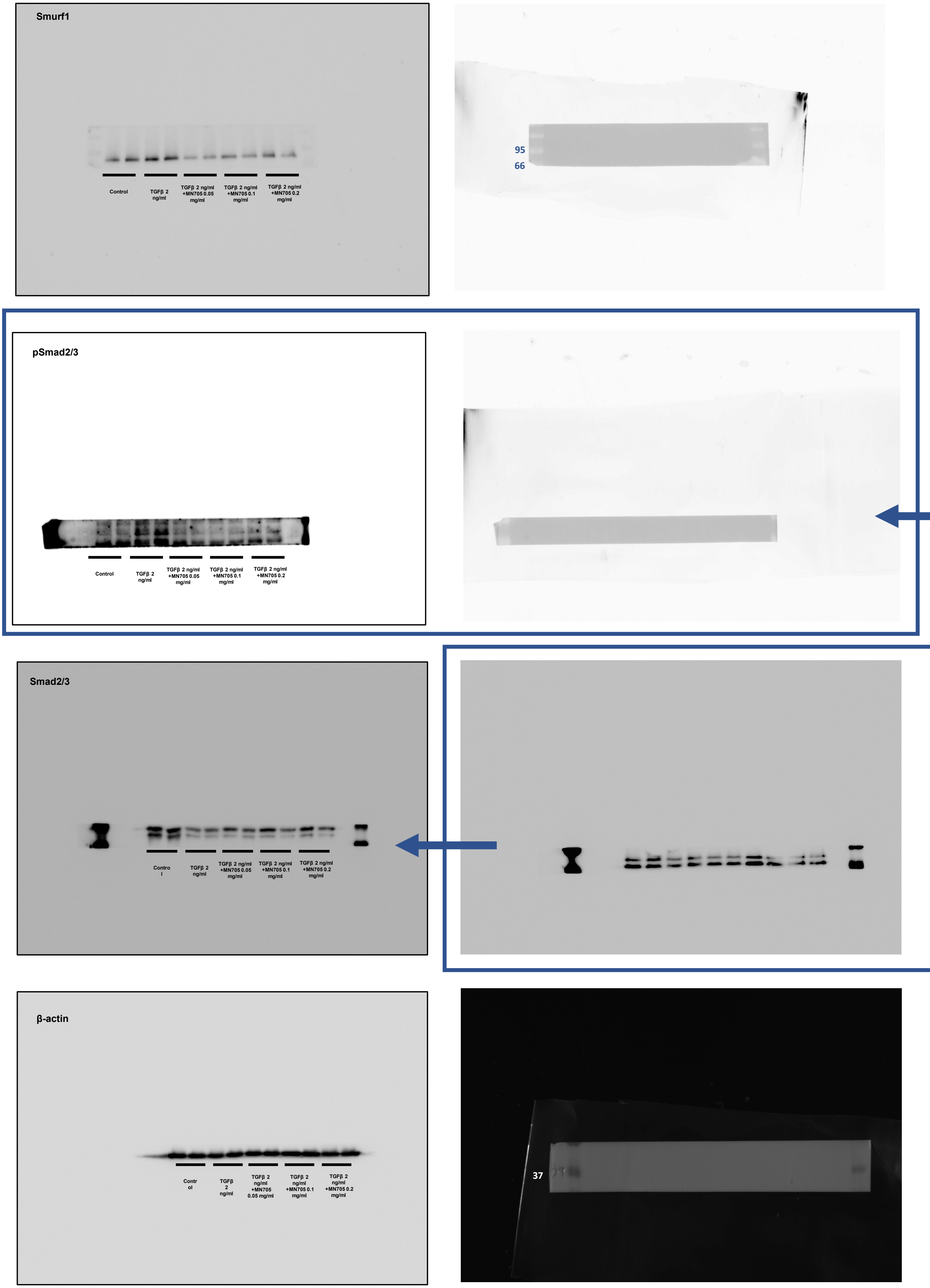

Smurf1
Control
TGFβ 2 ng/ml
TGFβ 2 ng/ml +MN705 0.05 mg/ml
TGFβ 2 ng/ml +MN705 0.1 mg/ml
TGFβ 2 ng/ml +MN705 0.2 mg/ml
95
66
pSmad2/3
Control
TGFβ 2 ng/ml
TGFβ 2 ng/ml +MN705 0.05 mg/ml
TGFβ 2 ng/ml +MN705 0.1 mg/ml
TGFβ 2 ng/ml +MN705 0.2 mg/ml
Smad2/3
Contro l
TGFβ 2 ng/ml
TGFβ 2 ng/ml +MN705 0.05 mg/ml
TGFβ 2 ng/ml +MN705 0.1 mg/ml
TGFβ 2 ng/ml +MN705 0.2 mg/ml
β-actin
Contr ol
TGFβ 2 ng/ml
TGFβ 2 ng/ml +MN705 0.05 mg/ml
TGFβ 2 ng/ml +MN705 0.1 mg/ml
TGFβ 2 ng/ml +MN705 0.2 mg/ml
37

Fig. 3 A/B(Only for statistics not used in main figure)

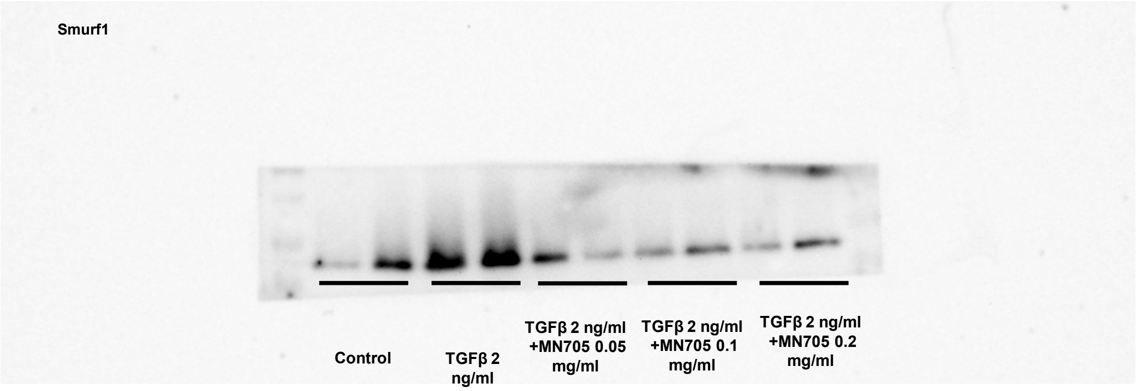

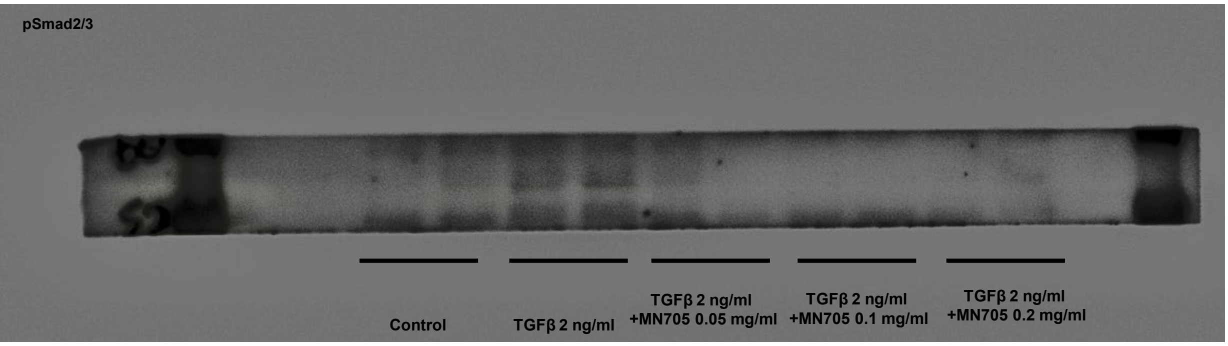

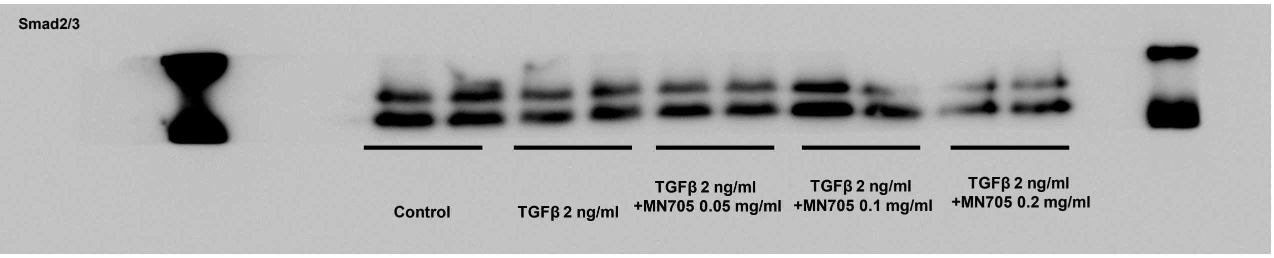

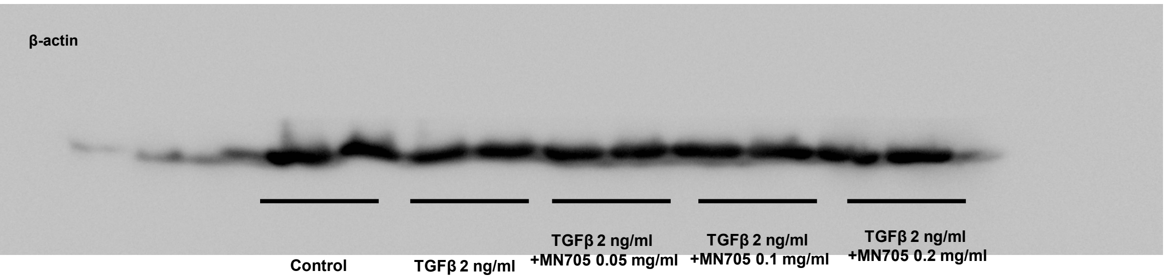

Fig. 3 C/D

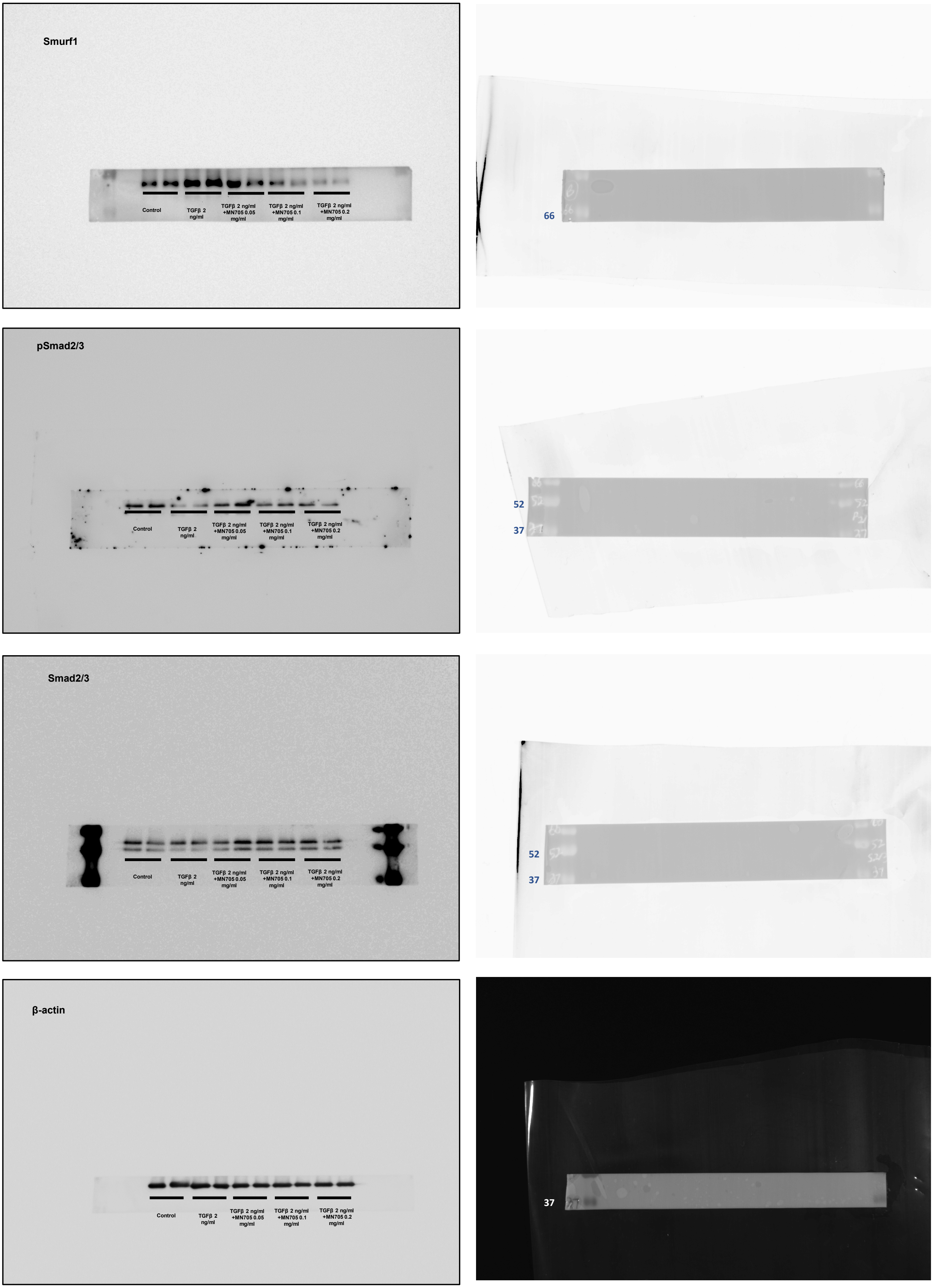

Smurf1
Control
TGFβ 2 ng/ml
TGFβ 2 ng/ml +MN705 0.05 mg/ml
TGFβ 2 ng/ml +MN705 0.1 mg/ml
TGFβ 2 ng/ml +MN705 0.2 mg/ml
66
pSmad2/3
Control
TGFβ 2 ng/ml
TGFβ 2 ng/ml +MN705 0.05 mg/ml
TGFβ 2 ng/ml +MN705 0.1 mg/ml
TGFβ 2 ng/ml +MN705 0.2 mg/ml
52
37
Smad2/3
Control
TGFβ 2 ng/ml
TGFβ 2 ng/ml +MN705 0.05 mg/ml
TGFβ 2 ng/ml +MN705 0.1 mg/ml
TGFβ 2 ng/ml +MN705 0.2 mg/ml
52
37
β-actin
Control
TGFβ 2 ng/ml
TGFβ 2 ng/ml +MN705 0.05 mg/ml
TGFβ 2 ng/ml +MN705 0.1 mg/ml
TGFβ 2 ng/ml +MN705 0.2 mg/ml
37

Fig. 3 C/D(Only for statistics not used in main figure)

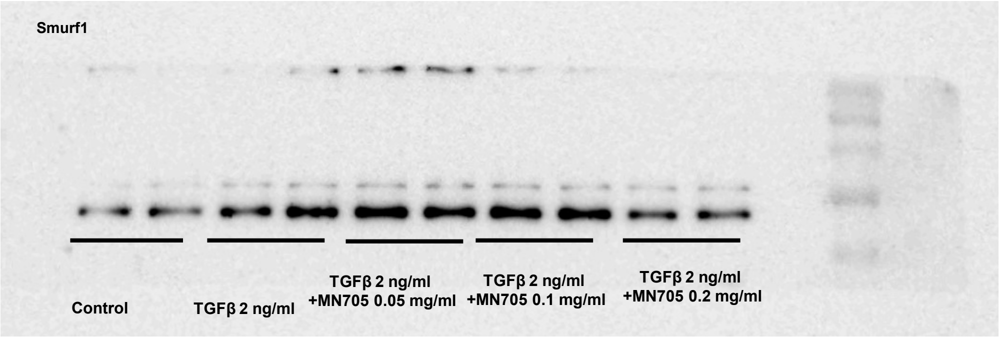

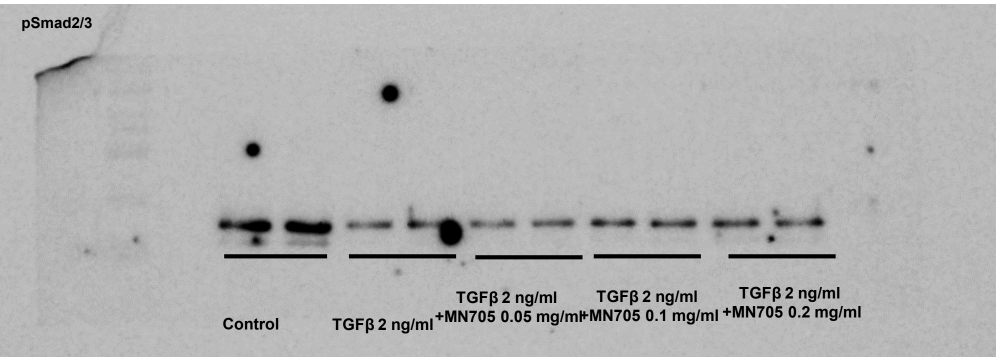

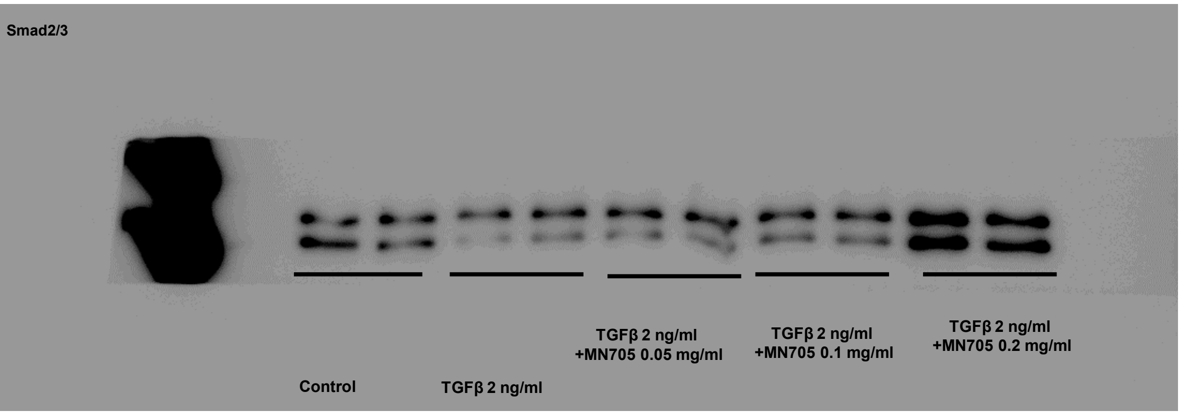

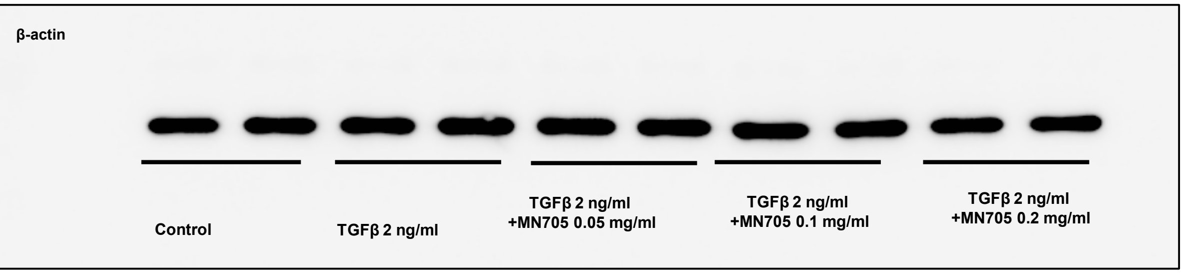

Fig. 4 B

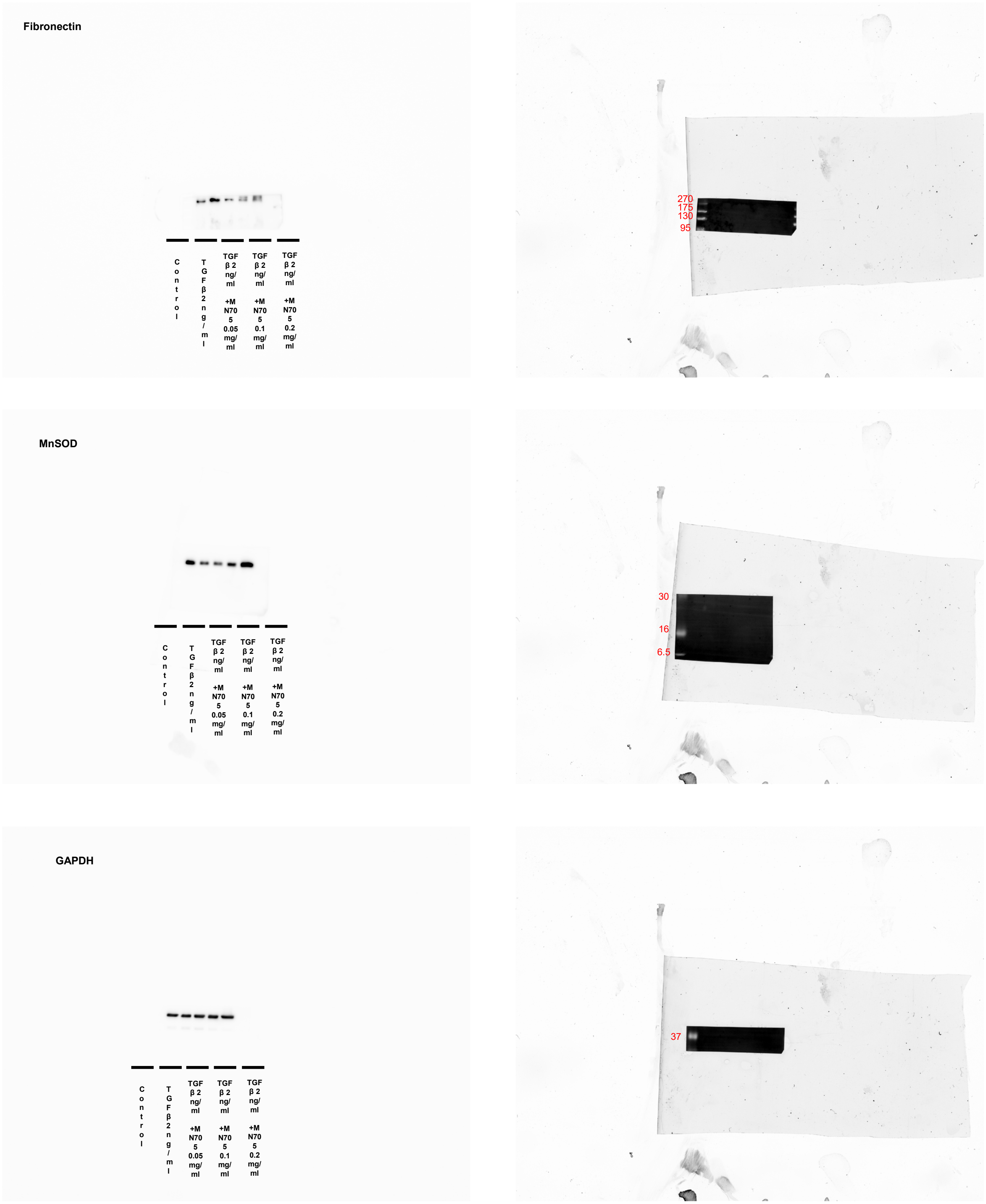
Fibronectin
Control
TGFβ2ng/ml
TGF β 2 ng/ ml +M N70 5 0.05 mg/ ml
TGF β 2 ng/ ml +M N70 5 0.1 mg/ ml
TGF β 2 ng/ ml +M N70 5 0.2 mg/ ml
270
175
130
95
MnSOD
Control
TGFβ2ng/ml
TGF β 2 ng/ ml +M N70 5 0.05 mg/ ml
TGF β 2 ng/ ml +M N70 5 0.1 mg/ ml
TGF β 2 ng/ ml +M N70 5 0.2 mg/ ml
30
16
6.5
GAPDH
Control
TGFβ2ng/ml
TGF β 2 ng/ ml +M N70 5 0.05 mg/ ml
TGF β 2 ng/ ml +M N70 5 0.1 mg/ ml
TGF β 2 ng/ ml +M N70 5 0.2 mg/ ml
37

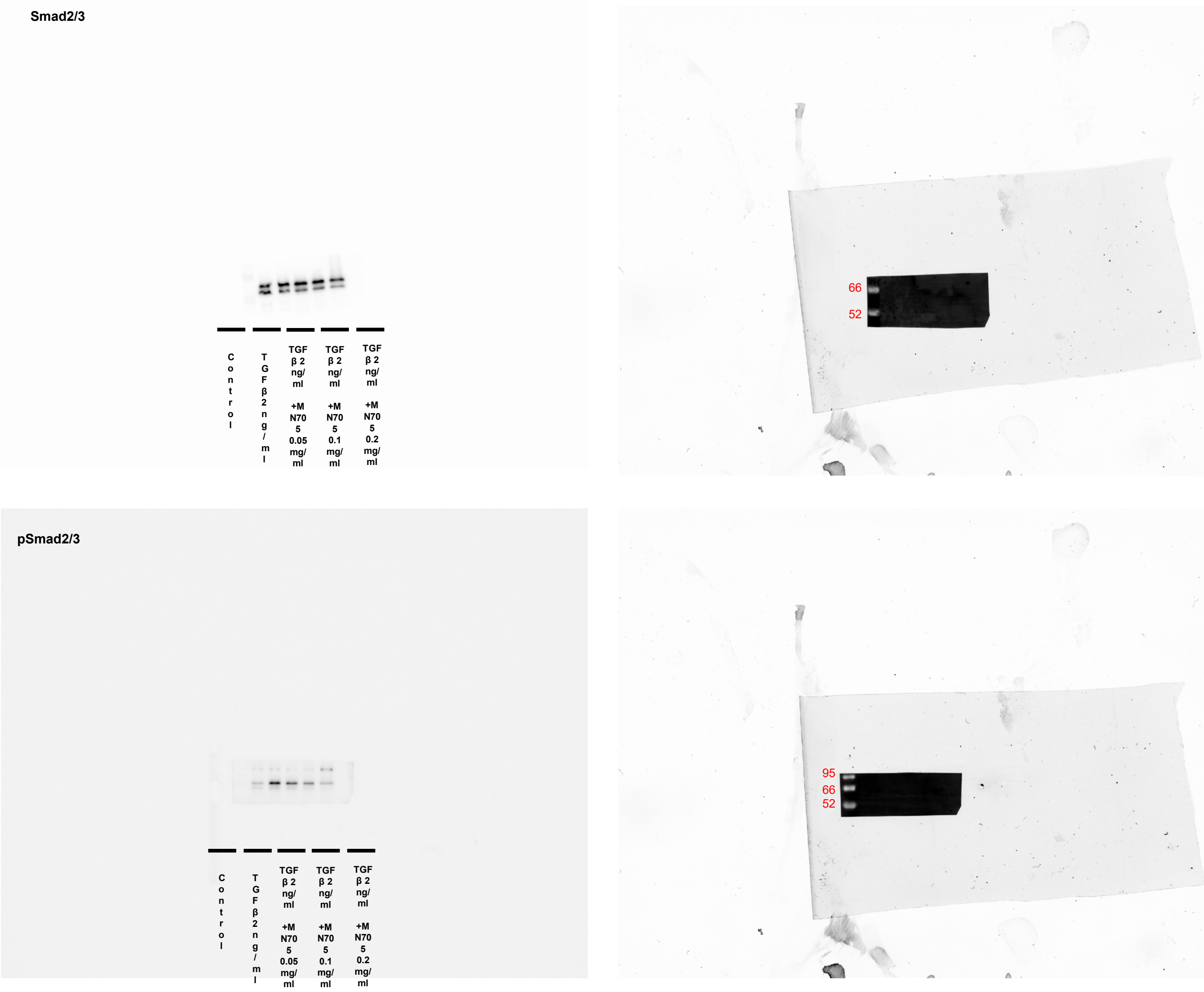
Smad2/3
Control
TGFβ2ng/ml
TGF β 2 ng/ ml +M N70 5 0.05 mg/ ml
TGF β 2 ng/ ml +M N70 5 0.1 mg/ ml
TGF β 2 ng/ ml +M N70 5 0.2 mg/ ml
66
52
pSmad2/3
Control
TGFβ2ng/ml
TGF β 2 ng/ ml +M N70 5 0.05 mg/ ml
TGF β 2 ng/ ml +M N70 5 0.1 mg/ ml
TGF β 2 ng/ ml +M N70 5 0.2 mg/ ml
95
66
52

Fig 4B ((Only for statistics not used in main figure1)

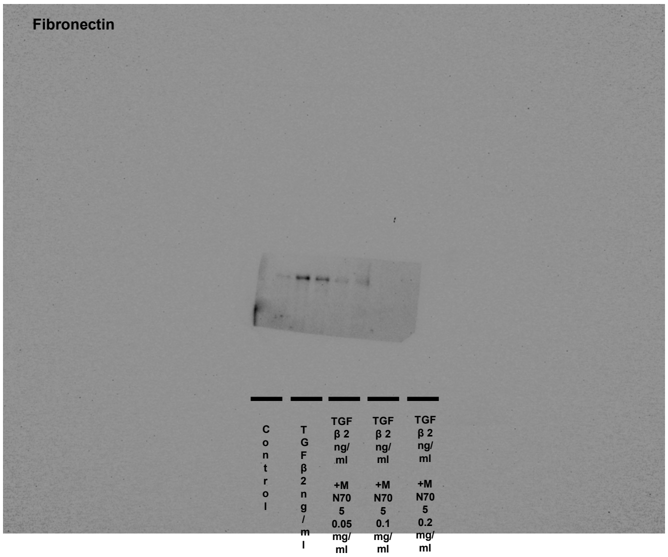

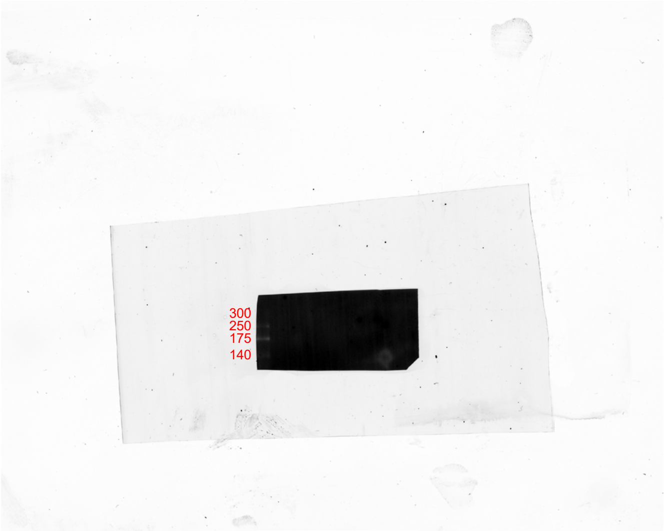

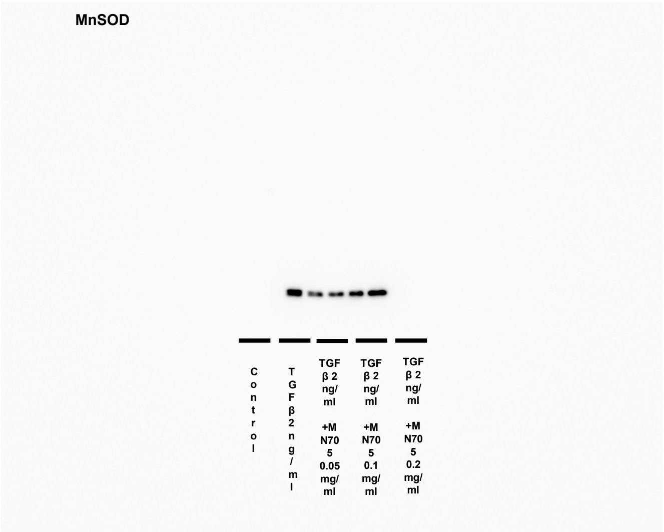

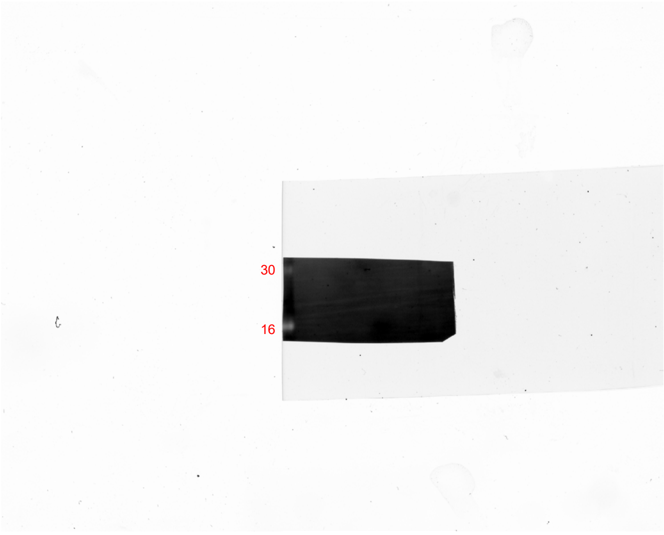

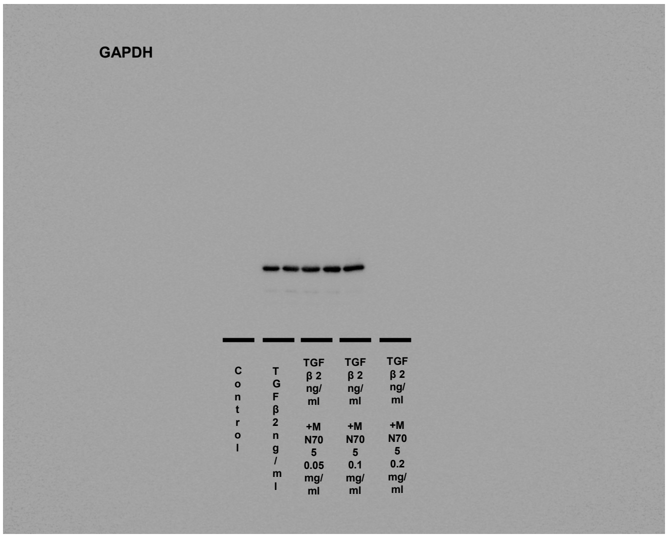

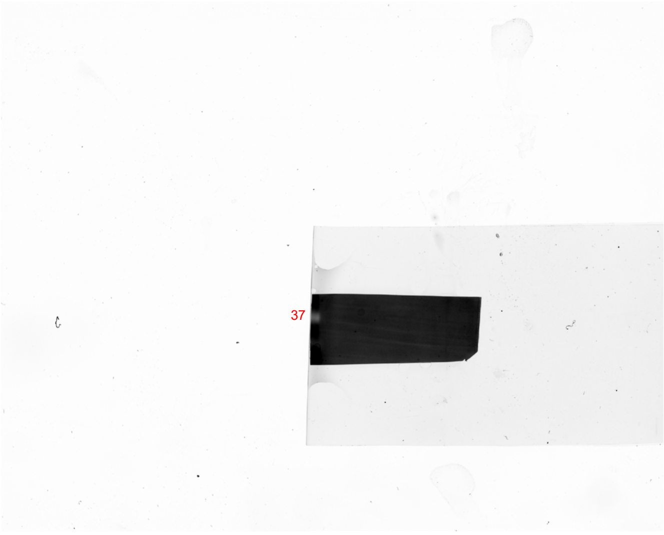

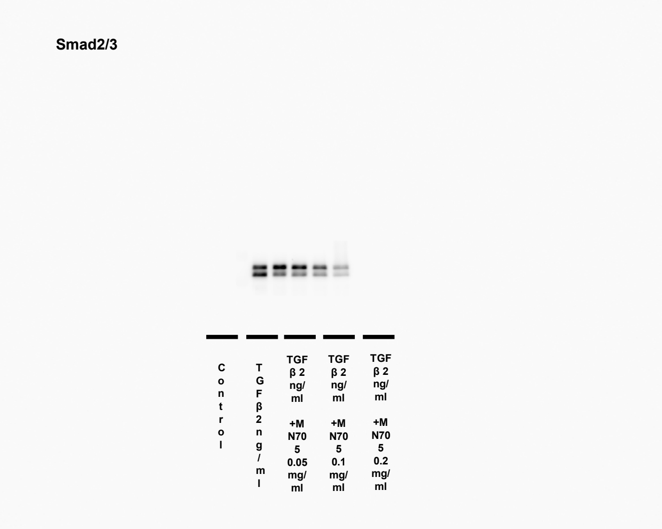
Smad2/3
Control
TGFβ2ng/ml
TGFβ 2 ng/ml +MN705 0.05 mg/ml
TGFβ 2 ng/ml +MN705 0.1 mg/ml
TGFβ 2 ng/ml +MN705 0.2 mg/ml

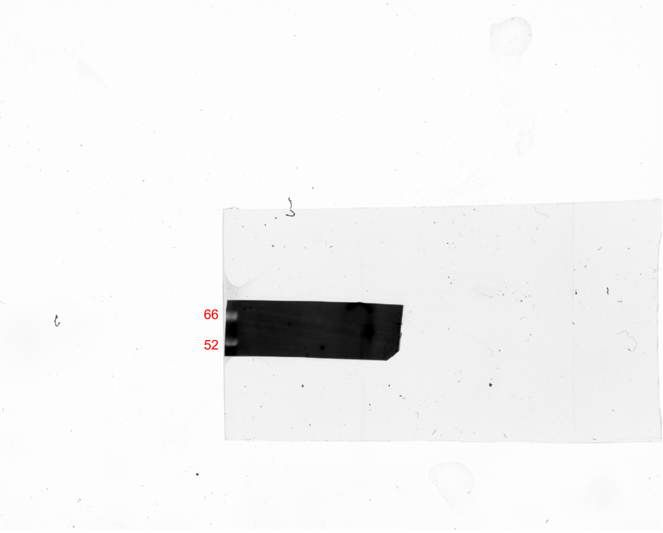
66
52

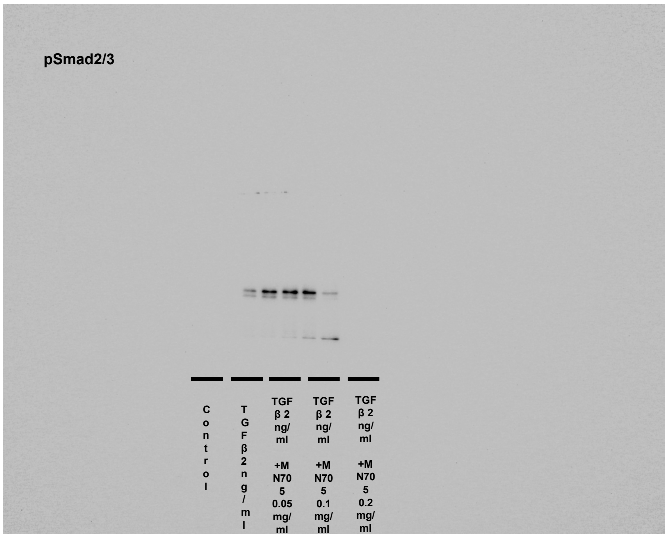
pSmad2/3
Control
TGFβ2ng/ml
TGFβ 2 ng/ml +MN705 0.05 mg/ml
TGFβ 2 ng/ml +MN705 0.1 mg/ml
TGFβ 2 ng/ml +MN705 0.2 mg/ml

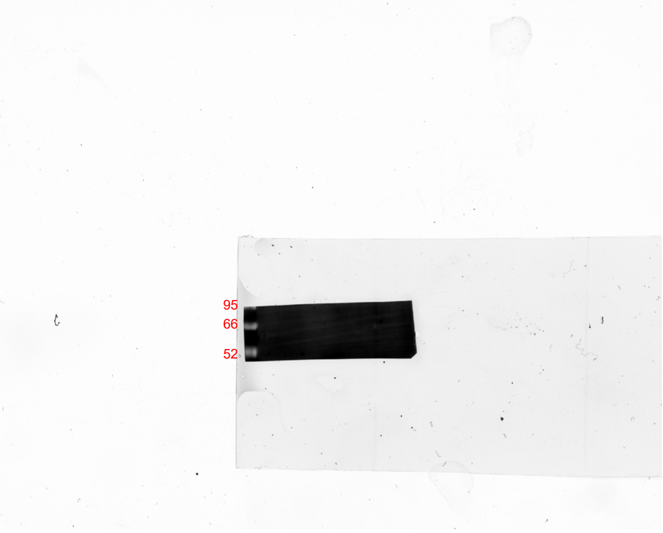
95
66
52

Fig 4B (only for statistics not used in main figure2)

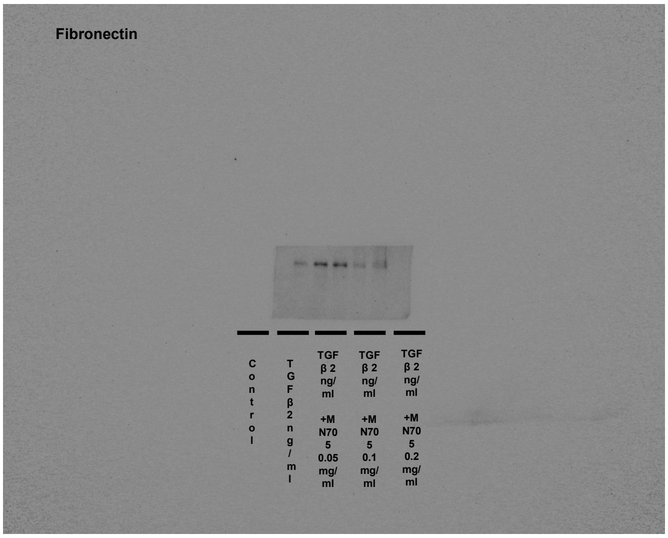

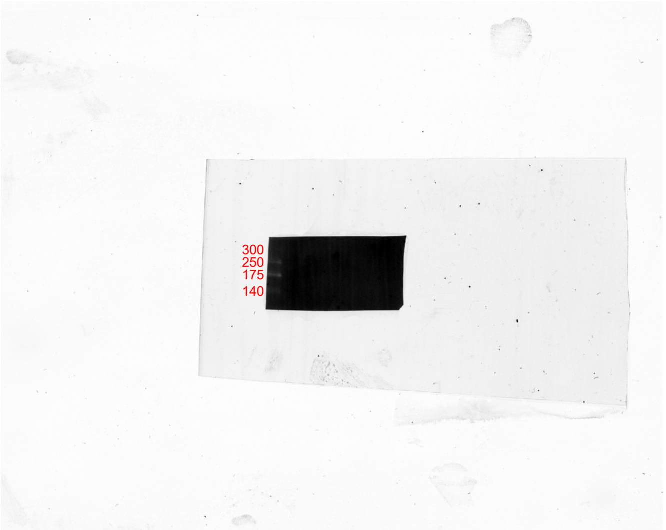

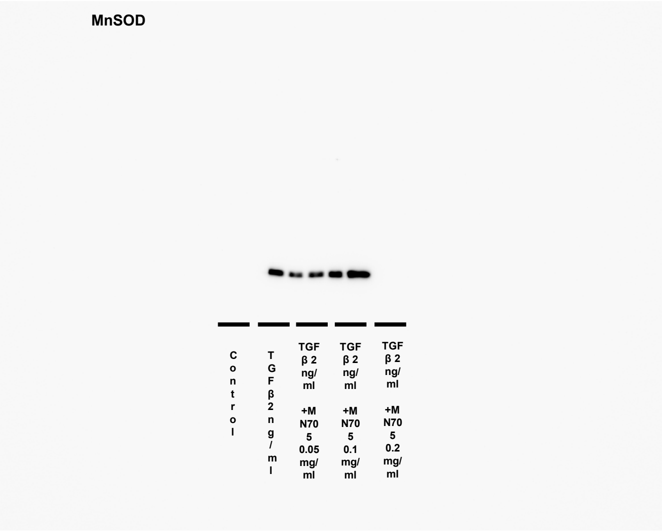

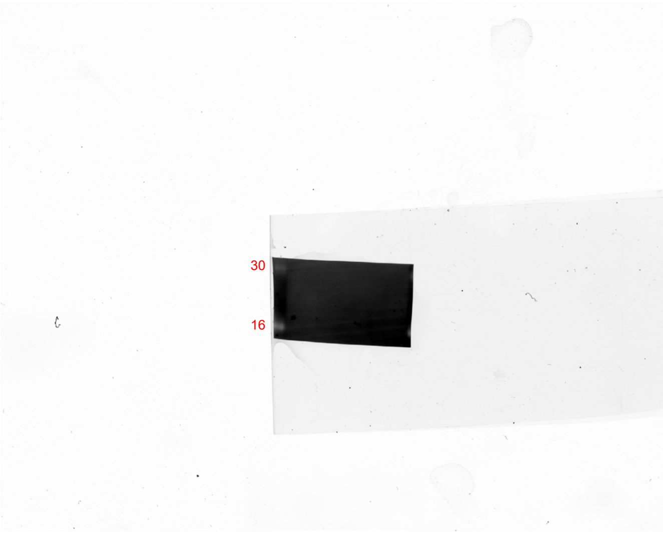

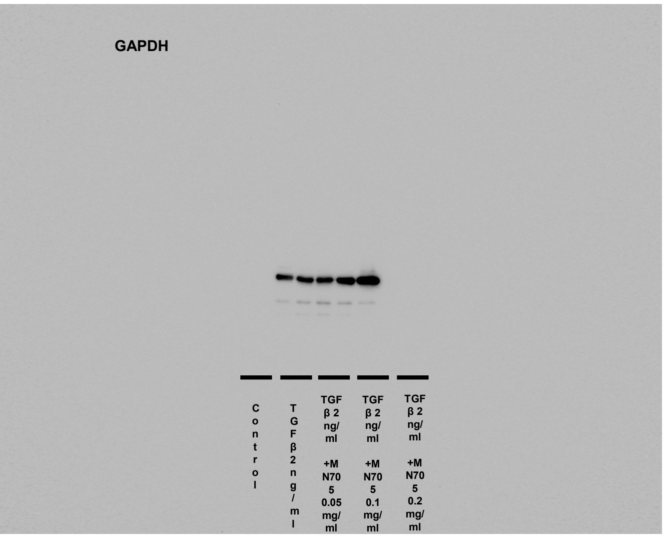

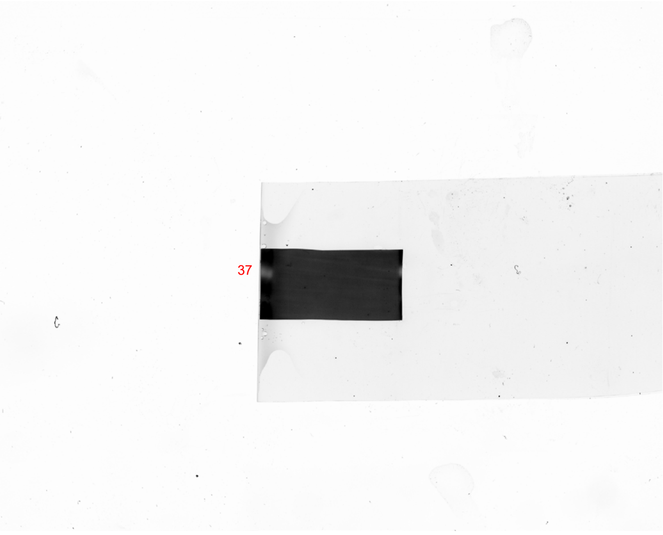

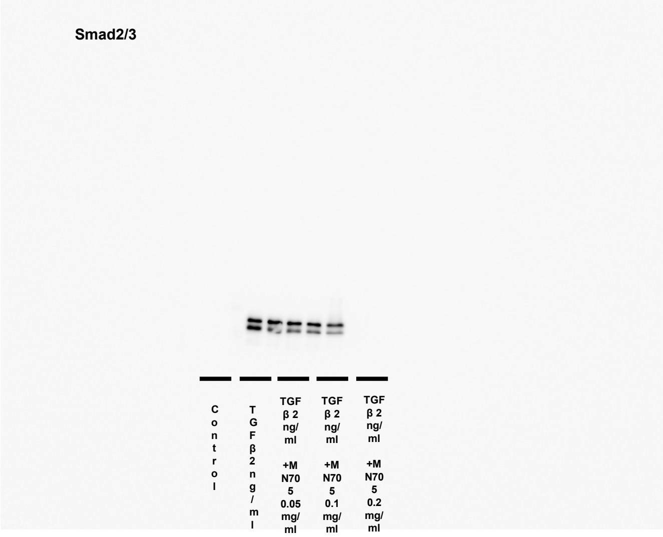
Smad2/3
Control
TGFβ2ng/ml
TGFβ 2 ng/ml +MN705 0.05 mg/ml
TGFβ 2 ng/ml +MN705 0.1 mg/ml
TGFβ 2 ng/ml +MN705 0.2 mg/ml

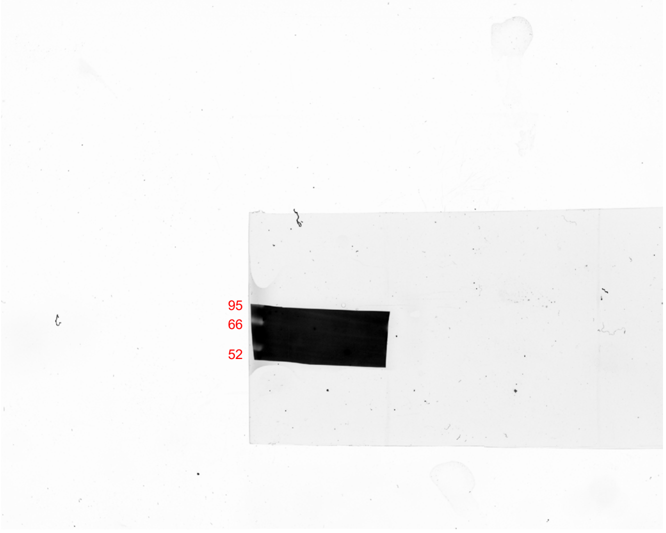
95
66
52

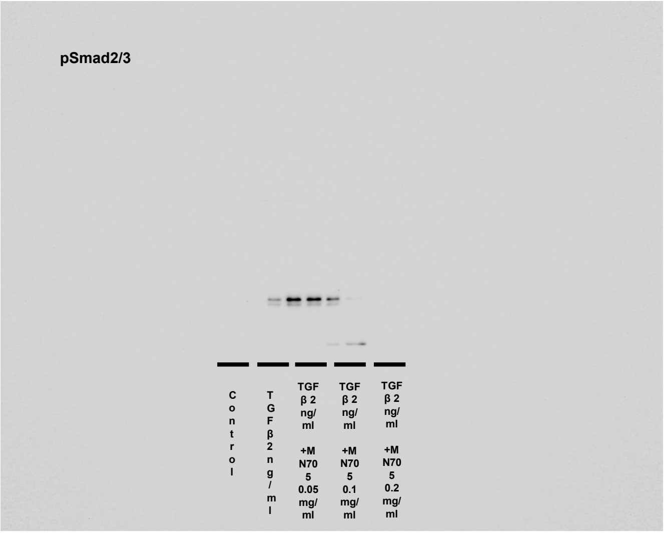
pSmad2/3
Control
TGFβ2ng/ml
TGFβ 2 ng/ml +MN705 0.05 mg/ml
TGFβ 2 ng/ml +MN705 0.1 mg/ml
TGFβ 2 ng/ml +MN705 0.2 mg/ml

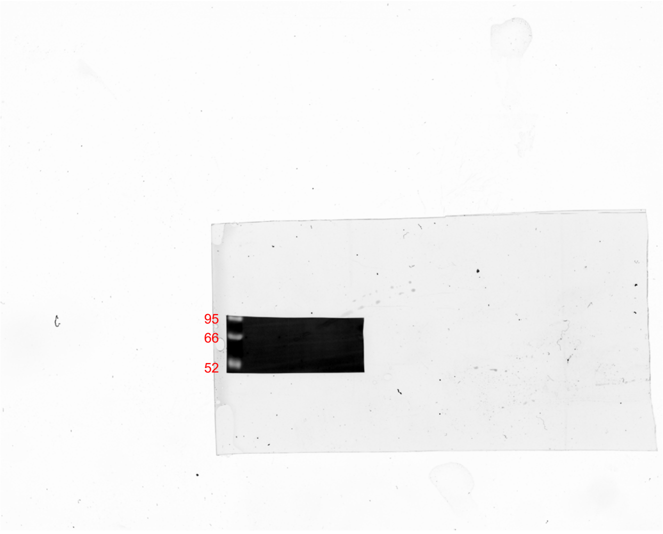
95
66
52

Fig. 6 A

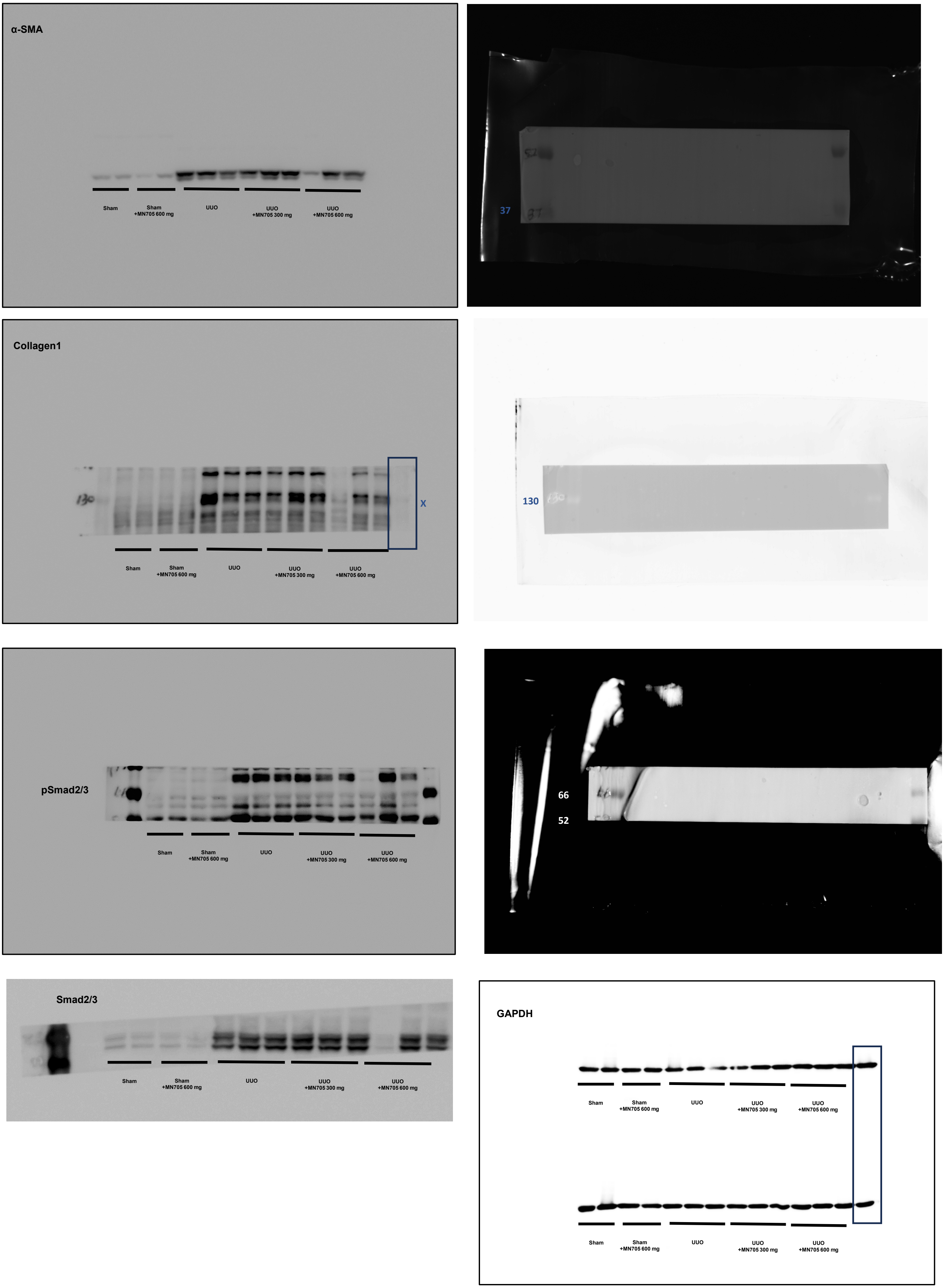
α-SMA
Sham
Sham +MN705 600 mg
UUO
UUO +MN705 300 mg
UUO +MN705 600 mg
37
Collagen1
130
X
130
pSmad2/3
66
52
Smad2/3
GAPDH

Fig. 6 A(Only for statistics not used in main figure)

α-SMA

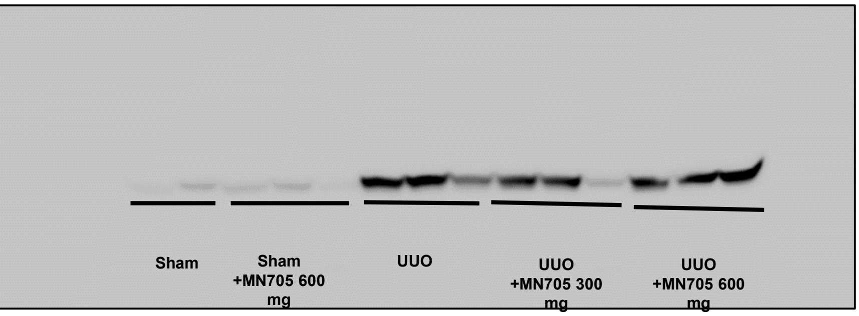

Collagen1

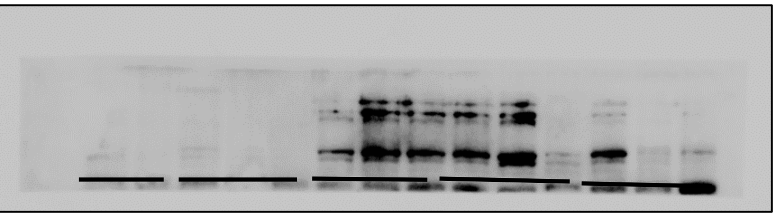

Sham

Sham
+MN705 600
mg

UUO

UUO
+MN705 300
mg

UUO
+MN705 600
mg

pSmad2/3

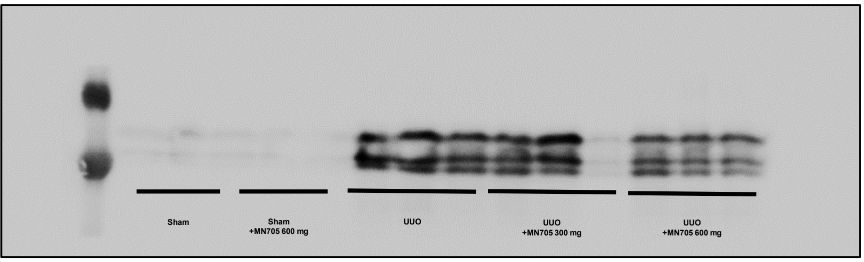

Smad2/3

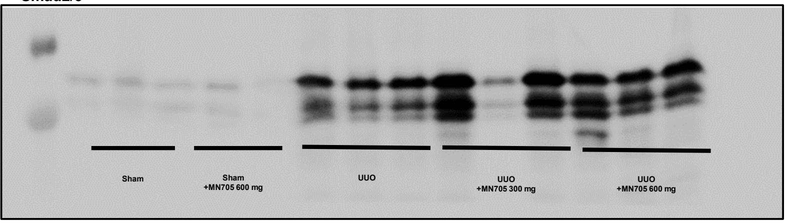

Fig. 7 A

MnSOD

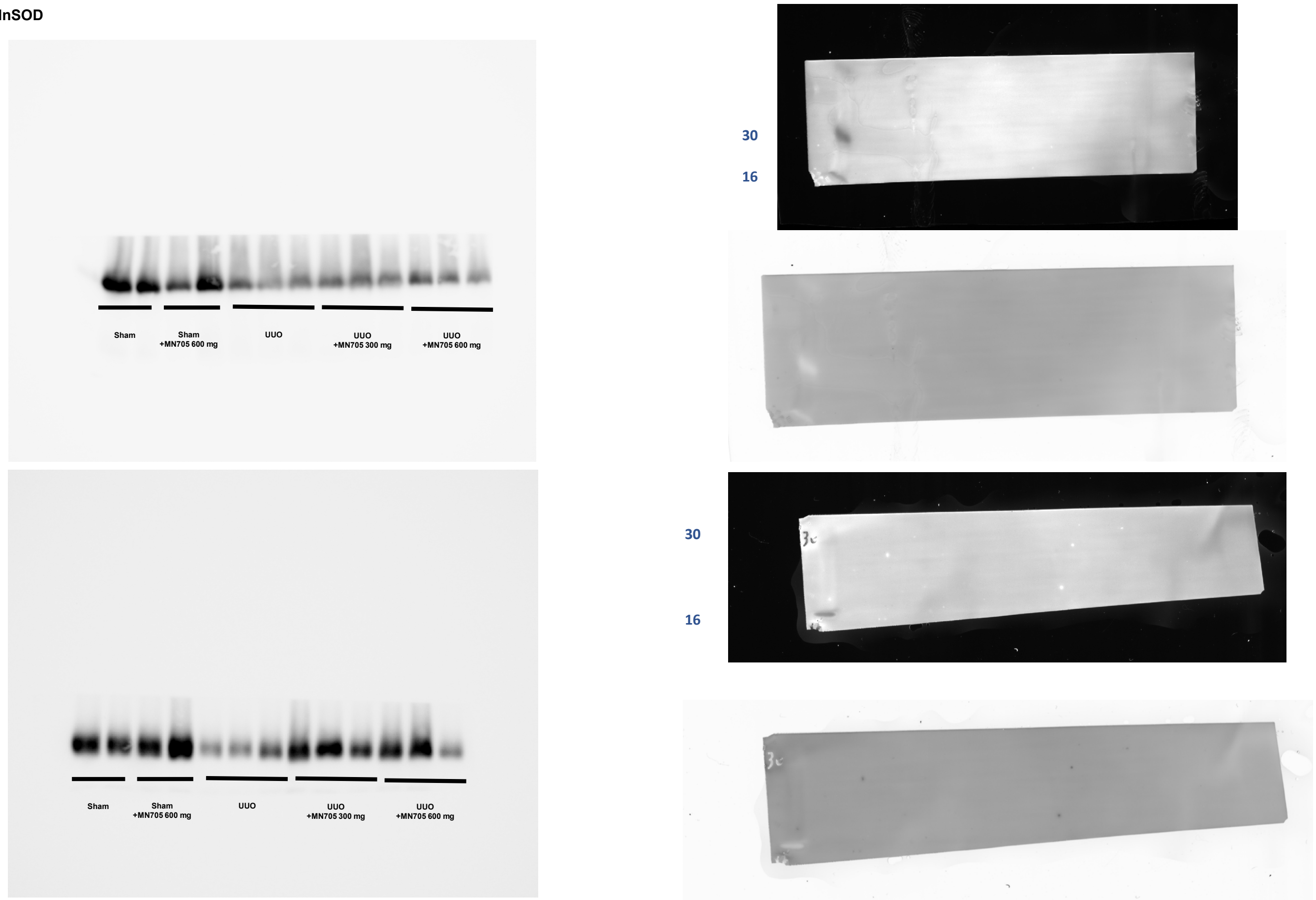

GAPDH

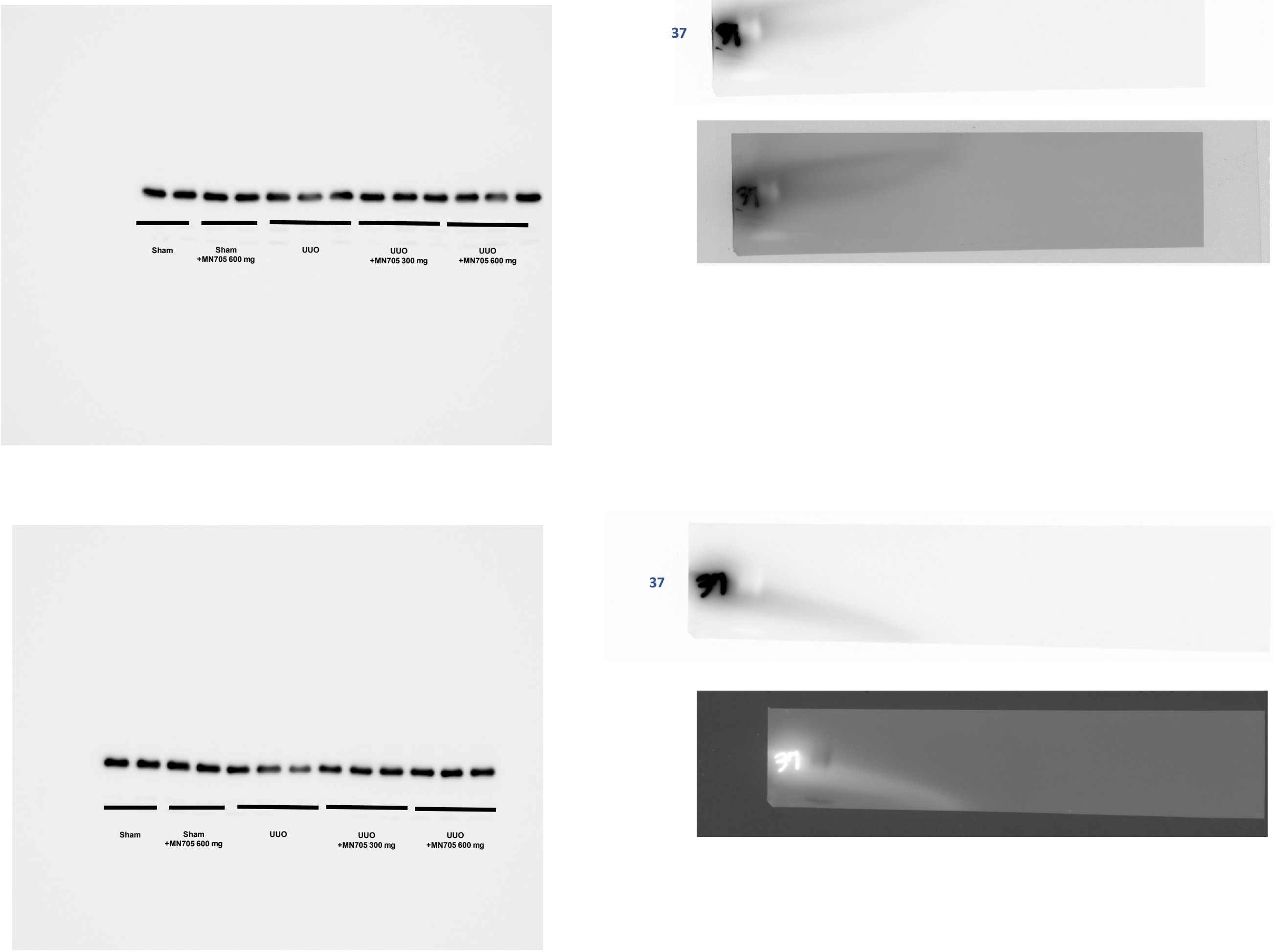

Supplements Fig1A

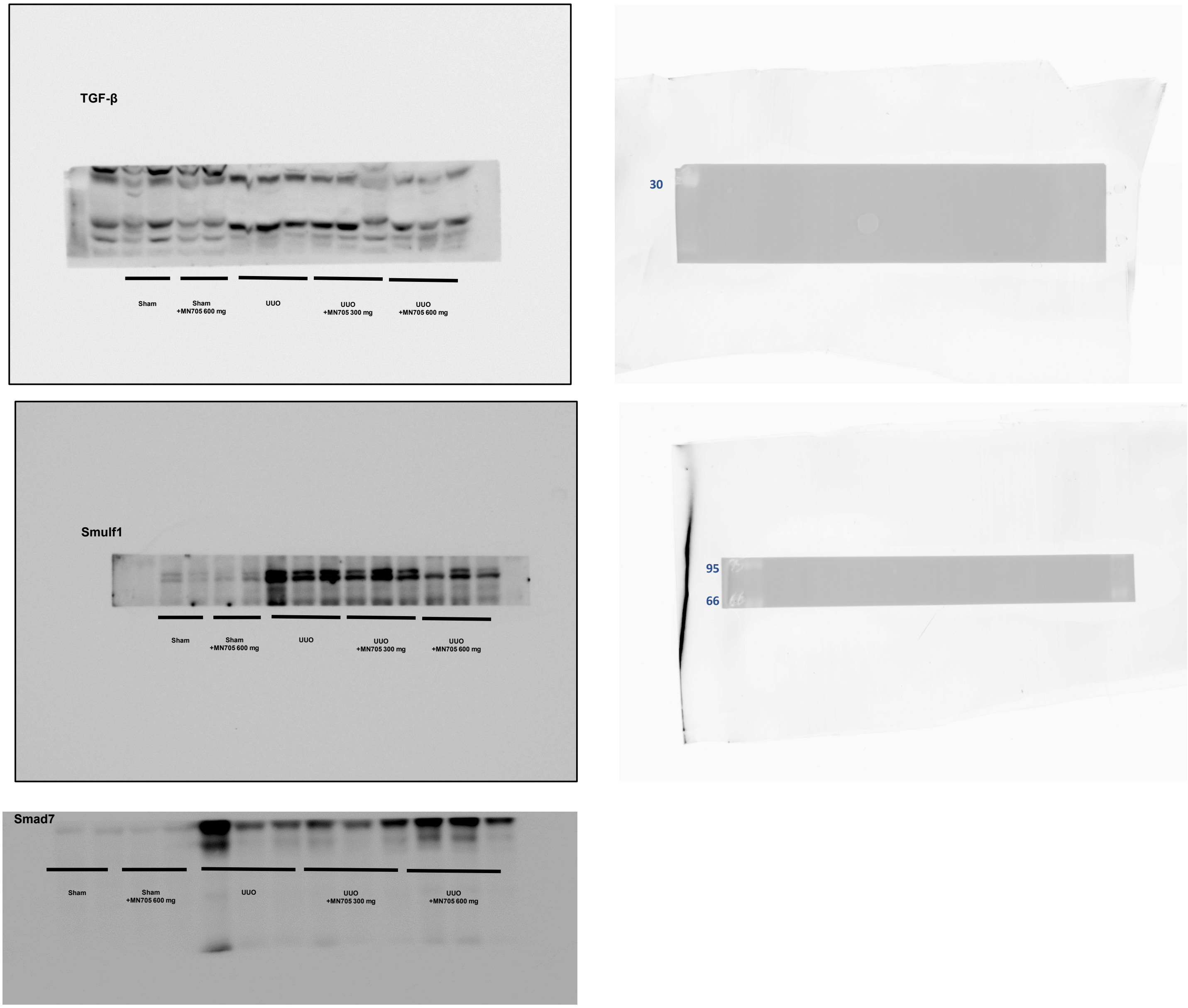

Supplement: S1 File — (PDF) [file pone.0321282.s002.pdf]
