# Supplementary material for: Rubus coreanus extract prevents kidney fibrosis through TGF-β/Smad pathway inhibition
Source: PLoS One. 2025 May 12;20(5):e0321282. doi: 10.1371/journal.pone.0321282 (PMC12068887; doi:10.1371/journal.pone.0321282)
Supplement: Attachment — Submitted filename: Rebuttal letter_MN705_240221_231210.docx [file pone.0321282.s004.docx]

**Comments from the Editor and Reviewers**

PONE-D-23-21717

Rubus coreanus extract attenuates kidney fibrosis through TGF-β/Smad pathway inhibition

PLOS ONE

**Editor comments:**

Dear Dr. Lee,

Thank you for submitting your manuscript to PLOS ONE. After careful consideration, we feel that it has merit but does not fully meet PLOS ONE’s publication criteria as it currently stands. Therefore, we invite you to submit a revised version of the manuscript that addresses the points raised during the review process.

==============================

Your manuscript has been reviewed by experts in the field. Their comments are attached for your view.

==============================

We look forward to receiving your revised manuscript.

Kind regards,

Qiang Ding, Ph.D.

Academic Editor

PLOS ONE

**Response:** The authors appreciate the decision and comments from the editor and reviewers. important comment. The authors have made the necessary improvements. In this revision, we have further confirmed the extent of oxidative damage (8-OHDG) and changes in antioxidant markers (MnSOD) in animal experiments. Furthermore, the authors have rectified the western blot measurement error and enhanced the image quality to ensure clear visibility of all numbers and letters when zoomed in. Thank you again for the time and opportunity to conduct further experimentation for this revision. Through the revision process, the authors were able to fill in the gaps and provide a more logical explanation for our findings.

The required files of a rebuttal letter (file labeled 'Response to Reviewers'), a marked-up copy of the manuscript (file labeled 'Revised Manuscript with Track Changes'), and an unmarked version of the revised paper without tracked changes ('Manuscript') were all prepared and uploaded. Financial disclosures are newly added to the editorial manager system. The authors have endeavored to follow the guidelines for resubmitting figure files. The authors ensure that this manuscript meets PLOS ONE's style requirements. The authors also attached the original uncropped and unadjusted images underlying all blot or gel results. In the cover letter, the authors noted that blot/gel image data are in Supporting Information. In the Methods section, we provided additional information regarding the experiments involving animals and ensured the authors included details on (1) methods of sacrifice, (2) methods of anesthesia and/or analgesia, and (3) efforts to alleviate suffering. The authors have used a professional scientific editing service to ensure that the English language is correct. The authors moved the ethics declaration, which appeared after the main text, to the methods section.

With this revision, we hope to have this study published in PLOS ONE. Thank you very much.

**Reviewers' comments:**

**Reviewer #1:**

The manuscript described potential protective effects of Rubus coreanus extract (RCEs) on kidney fibrosis through TGF-b/Smad pathway inhibition. They characterized dose-dependent effects of RCEs on TGF-b-stimulated fibrotic signaling in HK-2 cells in vitro and also in vivo animal model of kidney fibrosis. The strength of the manuscript is the identification on the impact of RCEs, that suppresses TGF-b/Smad2/Smurf profibrotic signaling pathway while induction of anti-fibrotic signaling of Smad7 and anti- oxidant MnSOD, that resulted into reduced fibrotic tissue response in UUO animal model of renal fibrosis. Overall, it is descriptive and there are several weaknesses that need to be further clarified. Followings are the specific comments:

1. In Figure 3, significantly different regulations of TGF-b-stimulated Smad signaling in HK-2 cells between acute and chronic exposures were noted. It may need additional discussion to explain why this happen and the impact of clinical application of RCEs.

**Response:** Thank you for the important comment. TGF-β signaling plays a crucial role in the process of fibrosis, a condition characterized by the excessive accumulation of extracellular matrix components in tissues. The canonical TGF-beta signaling pathway involves the activation of Smad proteins, particularly Smad2/3. The response of TGF-β/Smad signaling and phospho-Smad2/3 can differ between the early and late stages of fibrosis.

In response to tissue injury or inflammation, TGF-β is released and activated. The activated TGF-β binds to its receptors on the cell surface, leading to the phosphorylation of Smad2 and Smad3. Phosphorylated Smad2 and Smad3 form a complex with Smad4. This complex translocates into the nucleus, where it regulates the transcription of target genes involved in fibrosis, such as those encoding extracellular matrix proteins. In the early stages of fibrosis, the TGF-beta/Smad signaling pathway promotes the activation of fibroblasts, leading to the production and deposition of extracellular matrix components.

In late fibrosis, there is often sustained and prolonged activation of TGF-beta signaling. This persistent activation contributes to the chronicity of the fibrotic process. The continued activation of TGF-beta/Smad signaling leads to an ongoing synthesis of extracellular matrix components, surpassing the normal tissue repair requirements. A positive feedback loop may develop, where the deposited extracellular matrix promotes further TGF-beta activation, perpetuating the fibrotic process.

In animal studies, UUO causes continuous pressure damage to the kidneys, accelerating fibrosis. In cellular studies, TGF-beta-induced changes in Smad2/3 occur relatively quickly and are known to be transient. Inman et al. reported that the levels of nuclear phospho-Smad2 reach their highest point around 45 minutes after TGF-β stimulation and thereafter decrease after 5 hours (REF 1). Cui et al. investigated that TGF-β provoked a rapid SMAD2 phosphorylation within 15 min. Phospho-Smad2 reached its maximum after 1 hr both under normoxia and hypoxia. After 4 h, the level of p-Smad2 gradually declined under hypoxia compared to normoxia. These differences became significant after 8 hr (REF 2). Sustained receptor activation is necessary to sustain the presence of active Smads in the nucleus and to facilitate TGF-β-induced transcription.

- REF 1. Inman GJ, Nicolás FJ, Hill CS. Nucleocytoplasmic Shuttling of Smads 2, 3, and 4 Permits Sensing of TGF-β Receptor Activity. Mol Cell. 2002;10(2):283-94. doi: 10.1016/s1097-2765(02)00585-3
- REF 2. Cui W, Zhou J, Dehne N, Brüne B. Hypoxia induces calpain activity and degrades SMAD2 to attenuate TGFβ signaling in macrophages. Cell Biosci. 2015:5:36. doi: 10.1186/s13578-015-0026-x. eCollection 2015

In summary, TGF-β/Smad signaling is involved in the early initiation of fibrosis and contributes to the progression of fibrosis through sustained activation. Cellular experiments have shown that activation of phospho-Smad occurs between 45 minutes and 1 hour after stimulation and disappears after 4-5 hours. In this study, stimulation of HK-2 cells with TGF-β resulted in a decrease in Smad2/3 activation at 48 hours, but we observed a significant increase in the 6-hour early model. Therefore, to observe Smad activation in cellular models, it is necessary to use an early model within 6 hours. The understanding of these differences is crucial for developing *in vitro* model and targeted therapeutic strategies to intervene in the fibrotic process at different stages.

The authors added these explanation as follows: “(line 324-335) The response of TGF-β/Smad signaling and phospho-Smad2/3 can differ between the early and late stages of fibrosis. In the early stages of fibrosis, the TGF-β/Smad signaling pathway promotes the activation of fibroblasts, leading to the production and deposition of extracellular matrix components. In late fibrosis, there is often sustained and prolonged activation of TGF-β signaling. This persistent activation contributes to the chronicity of the fibrotic process. In animal studies, UUO causes continuous pressure damage to the kidneys, accelerating fibrosis. Cellular experiments have shown that activation of phospho-Smad2 occurs between 45 minutes and 1 hour after stimulation and disappears after 4-5 hours [33, 34]. In this study, stimulation of HK-2 cells with TGF-β resulted in a decrease in Smad2/3 activation at 48 hours, but we observed a significant increase in the 6-hour early model. Therefore, to observe Smad activation in cellular models, it is necessary to use an early model within 6 hours. The understanding of these differences is crucial for developing *in vitro* model and targeted therapeutic strategies to intervene in the fibrotic process at different stages.”

2. Quantitative Western blot evaluations on TGF-b-stimulated Smad2/3 were not well established based on the band pattern, that need to be consistently applied for accurate quantitative measurement.

**Response:** Thank you for the valuable comment. Following the reviewer's comments, we have re-quantified the western blot gels for TGF-beta/Smad pathway-related markers (Fig. 3). We apologize for the inaccurate results and figures. The text description for Fig. 3 has also been modified as follows: “(line 192-200) TGF-β treatment for 6-h increased the expression of phospho-Smad2/3 (1.64 ± 0.06 fold changes compared to control, *P* < 0.001) and Smad ubiquitination regulator 1 (Smurf1) (1.46 ± 0.15 fold changes compared to control, *P* < 0.001), which was significantly reduced by MN705 treatment (Fig. 3A, B). In the TGF-β-treated (48-h) HK-2 cell *in vitro* model, Smurf1 expression increased and significantly attenuated by MN705 treatment (1.70 ± 0.35, 1.48 ± 0.24, 0.65 ± 0.24, and 0.18 ± 0.19 fold changes compared to control, overall *P* < 0.001; TGF-β 2 ng/ml with vehicle, MN705 0.05 mg/ml, 0.01 mg/ml, and 0.02 mg/ml, respectively); however, phospho-Smad2/3 decreased with prolonged exposure to TGF-β and the decrease after MN705 treatment was not significant (Fig. 3C, D).”

3. RCEs are very complex natural product. It may help to use specific component of the complex to characterize in vivo and in vitro effect. Al least some discussions on this aspect.

**Response:** Thank you for your valuable suggestion. Rubus coreanus, a natural substance, consists of a variety of ingredients. Analyzing the constituents of Rubus coreanus and identifying those that have antifibrotic effects is important, but it requires additional experiments that are beyond the scope of this study. Therefore, the authors added the following information from the literature review to the discussion section: “(line 286-The constituents of Rubus coreanus can vary, but it generally contains a range of bioactive compounds that contribute to its potential health benefits. The representative components of Rubus coreanus include polyphenols, vitamins, and minerals. Polyphenolic compounds, such as anthocyanins, flavonoids, and ellagic acid have been widely studied for their antioxidant properties. Rubus coreanus may contain various vitamins and minerals, such as vitamin C and manganese, which also contribute to its antioxidant capacity. The antioxidant properties of Rubus coreanus can play a crucial role in neutralizing free radicals and reducing the production of reactive oxygen species and pro-inflammatory cytokines, thereby inhibiting fibrogenesis [21].”

“(line294-302) There has been research investigating the potential role of antioxidants in treating kidney fibrosis in experimental studies. Antioxidants are studied for their ability to reduce oxidative stress, inflammation, and cellular damage, which are associated with the development of fibrosis. N-Acetylcysteine (NAC) is a precursor to glutathione, a powerful endogenous antioxidant. Some studies have investigated the potential of NAC and it’s anti-oxidative effect in ameliorating kidney fibrosis [22]. Resveratrol is a polyphenol found in certain foods, including red grapes and berries. Chowdhury et al. demonstrated that resveratrol treatment reduced kidney fibrosis in a high-fat diet rat model, possibly through its antioxidant and anti-inflammatory effects [23].”

“(line 348-350) While these studies suggest a potential therapeutic role for antioxidants in kidney fibrosis, it's essential to approach the findings with caution.” The translation of experimental results to clinical applications requires further research, and “(line 350-352) the effectiveness of antioxidants may vary depending on the specific conditions and causes of kidney fibrosis.” It's important to note that while Rubus coreanus shows promise in terms of its antioxidant properties, “(line 351-352) further research is needed to fully understand the specific mechanisms and potential health benefits associated with Rubus coreanus.”

- [22] Shen Y, Miao NJ, Xu JL, Gan XX, Xu D, Zhou L, et al. N-acetylcysteine alleviates angiotensin II-mediated renal fibrosis in mouse obstructed kidneys. Acta Pharmacol Sin. 2016;37(5):637-44. doi: 10.1038/aps.2016.12
- [23]. Chowdhury FI, Yasmi T, Akter R, Islam MN, Hossain MM, Khan F, et al. Resveratrol treatment modulates several antioxidant and anti-inflammatory genes expression and ameliorated oxidative stress mediated fibrosis in the kidneys of high-fat diet-fed rats. Saudi Pharm J. 2022;30(10):1454-1463. doi: 10.1016/j.jsps.2022.07.006

4. They provide only preventive effect, testing therapeutic effect would be more promising for clinical application.

**Response:** Thank you for the insightful comment. In general, renal fibrosis is an intractable disease, making it difficult to prove the efficacy of treatments. Furthermore, natural products are typically used for health promotion and disease prevention rather than as therapeutic agents. In light of these considerations, the researchers concluded that the null result was likely to be confirmed if the MN705 used in this study was used for therapeutic purposes. In response to the reviewer's good comments, we have added the following additional statement to the discussion: "(line 352-354) In addition, this study only confirmed the preventive effect of Rubus coreanus on anti-fibrosis. Further experiments and clinical studies are necessary to determine if the Rubus coreanus has a therapeutic effect on kidney fibrosis.”

**Reviewer #2**:

While the paper presents an interesting research for kidney fibrosis, there are significant questions to be addressed before the conclusion and mechanism can be appropriately supported.

1. What is the rationale for using TGFb in cells, while the in vivo model using Rubus coreanus which has anti-oxidative effects?

2. There is a disconnection between the mechanism investigated and results. Results are mainly fibrosis, nothing to do with anti-oxidation effects in cells and animal UUO models.

3. Cell results and animal results are not comparable.

**Response:** Thank you for the valuable comment. In the original submission, the researchers wanted to show that the MN705 compound had antifibrotic effects in animal models and to explain how these effects happened in the TGF-β-challenged HK-2 kidney cellular models with TGF- β/Smad pathway and oxidative stress. We could confirm that MN705 attenuated the kidney cellular fibrosis response and increased antioxidants in the 48-hour model (Fig. 2). However, we could not confirm the expected Smad pathway change in 48-hour model. This led the researchers to check the 6-hour early model, thinking that the Smad pathway that induces fibrosis might act earlier than 48 hours. The 6-hour model showed what was expected: it proved the changes in the Smad pathway (increased Smurf1 and phospho-Smad2/3 decrease after MN705) that could explain why renal cell fibrosis was prevented after MN705 treatment.

A substantial research supports the role of oxidative damage as a cause of kidney fibrosis. The purpose of *in vitro* experiment was not to prove that oxidative damage causes kidney fibrosis but to create a model of kidney cell fibrosis that can mimic the animal model of kidney fibrosis. Therefore, TGF-β was challenged to HK-2 cells.

In our original submission, we did not find any evidence of anti-oxidation or changes in markers of oxidative stress along with improvement of fibrosis in the animal *in vivo* model. To correct this discrepancy, we investigated the changes in MnSOD and 8-OHDG in the animal model (Fig. 6). Following MN705 treatment, MnSOD (anti-oxidative enzyme), which was reduced in UUO, showed a tendency to increase. The oxidative stress of 8-OHDG was significantly increased in UUO and significantly decreased after MN705 treatment. These results were described as follows: “(line 236-243) In addition, we also identified changes in anti-oxidative enzymes and oxidative stress in an *in vivo* model. The expression of MnSOD decreased significantly after the UUO operation (Fig 6A). In the MN705 treatment group, reduced MnSOD expression showed a relatively increased response with no statistical significance (Fig 6C). We measured the level of oxidative stress using 8-OHDG immunohistochemical staining. The UUO group showed significantly increased tissue expression levels of 8-OHDG (Fig 6B, D). After MN705 treatment, the MN705 treatment group showed a significant decrease in oxidative stress expressed with 8-OHDG.”

4. Although there is a dose response, not sure how the doses were selected, or any unwanted effects. What will be lowest dose to be effective?

**Response:** Thank you for the valuable comment. Although not detailed in the paper, various studies were consulted to determine the animal dose of Rubus coreanus. In the studies described below, we found that doses of 50-500 mg/kg/day were commonly used as therapeutic doses. Lee et al. reported anti-obesity effects at a therapeutic dose of 300 mg/kg/day, and Kim et al. confirmed anti-dyslipidemic effects at 250 mg/kg/day. In general, antifibrotic effects are not easy to prove experimentally. Therefore, we decided to use a relatively high dose of 300 mg/kg/day as the primary dose to prevent the null results. In addition, we set up a 600 mg/kg/day high dose group in this experiment to check the dose response of antifibrotic effects and other unexpected side effects.

- Om et al. Molecules. 2016;21(1):65. doi: 10.3390/molecules21010065
- Oh et al. Evid Based Complement Alternat Med. 2016:2016:4357656. doi: 10.1155/2016/4357656
- Jeong et al. Int J Obes (Lond). 201;39(3):456-64. doi: 10.1038/ijo.2014.155
- Jung et al. J Med Food. 2007;10(4):689-93. doi: 10.1089/jmf.2006.006
- Lee et al. Kor J Food Nutrition. 2018;31(2):242-51. doi:10.9799/ksfan.2018.31.2.242
- Kim et al. Nutrients. 2018 1;10(12):1846. doi: 10.3390/nu10121846.

5. What about time responses in animal models?

**Response:** Thank you for the critical comment. As the reviewer notes, the response of MN705 may be different in animal studies, as shown in cellular experiments with early versus late stages of fibrosis. Recently, many researchers in the nephrology field have shown great interest in exploring the pathophysiological characteristics of a new disease entity called the AKI-to-CKD transition. This kidney disease model progresses to fibrosis after an acute kidney injury. Researchers have widely used the unilateral ischemia-reperfusion injury model as an animal model of the AKI-to-CKD transition. However, this study explored the anti-fibrotic effect of MN705 in a renal fibrosis model and its mechanism. In the UUO model, fibrosis is typically identified between days 7 and 14; in this study, we observed fibrosis specifically at day 7. It would be interesting to explore the change in fibrosis and its molecular indications, the TGF-β/Smad pathway, and oxidative damage in the kidney at a shorter time after UUO surgery. However, conducting another animal study is necessary to explore the early fibrosis response, which goes beyond our initial research goals. We ask for the reviewer’s generous understanding of not being able to reflect the reviewers' valuable comments on another animal experiment. The authors added these explanations of limitation on the discussion part of as follows: “(line 354-357 At the last, the anti-fibrotic impact of Rubus coreanus was only observed in the 7-day model of the UUO mouse. Therefore, it is worthwhile to investigate if this anti-fibrotic effect persists over shorter or longer time intervals.”

6. Image quality needs to be improved.

**Response:** Thank you for the valuable comment. As the reviewer pointed out, some of the pictures were poorly resolved, and letters and numbers were difficult to discern. We took the reviewer's advice and printed the figures at a higher resolution throughout. For Fig. 1., we increased the size and resolution of the graph so that the numbers and letters in the graph are clearly visible. We also made additional efforts to enhance the quality of the figures throughout.

7. Mechanism involved is not clear, or at least not supported by the current data for the effect of Rubus coreanus.

**Response:** Thank you for the valuable comment. As pointed out by the reviewer, a limitation of this study is that it did not directly confirm the modulation of TGF-β/Smad signaling and antifibrotic effects by antioxidants. It's important to note, though, that this study is the first to confirm that Rubus coreanus can help prevent renal fibrosis. It also suggests that antioxidant effects and controlling TGF-β/Smad signaling may play a role in how this works. In addition, Fig. 6 demonstrates that the antioxidant effect was also observed in in vivo animal experiments, which were not included in the original submission. The strengths and limitations of this study with the mechanism of anti-fibrosis are included at the end of the discussion as follows: “(line 336-357) Although the direct mechanism of regulation of TGF-β/Smad signaling by antioxidants was not unveiled in this study, treatment with Rubus coreanus effectively regulated TGF-β/Smad signaling in both *in vitro* and *in vivo* models and alleviated kidney fibrosis. There has been some evidence that support anti-oxidation via phenolic compounds of phytochemicals can attenuate kidney fibrosis via Smad pathway. Berberine (isoquinoline alkaloid; found in Coptidis Rhizoma and Cortex Phellodendri) had anti-fibrosis effects via inhibition of Smad2/3 in streptozocin-induced diabetic kidney disease model [35]. Dioscin (steroid saponin; found in Dioscoreae rhizome) showed anti-oxidative and anti-fibrosis effects with inhibition of Smad3 and activation of Smad7 in 10% fructose-fed mice model [36]. Sinomenine (Alkaloid; found in Sinomenium acutum) also had anti-oxidative and anti-fibrosis effects with inhibition of Smad3 in UUO mice model and TGF-β/H_2_O_2_-induced *in vitro* fibrosis model [37]. This study has the unique advantage of being the first to show the antifibrotic properties of Rubus coreanus in kidney fibrosis. Additionally, it proposes that the modulation of TGF-β/Smad signaling, along with the anti-oxidative effects, is the underlying mechanism of anti-fibrosis. While these studies suggest a potential therapeutic role for antioxidants in kidney fibrosis, it's essential to approach the findings with caution. The effectiveness of antioxidants may vary depending on the specific conditions and causes of kidney fibrosis. Further research is needed to fully understand the specific mechanisms and potential health benefits associated with Rubus coreanus. In addition, this study only confirmed the preventive effect of Rubus coreanus on anti-fibrosis. Further experiments and clinical studies are necessary to determine if the Rubus coreanus has a therapeutic effect on kidney fibrosis. At the last, the anti-fibrotic impact of Rubus coreanus was only observed in the 7-day model of the UUO mouse. Therefore, it is worthwhile to investigate if this anti-fibrotic effect persists over shorter or longer time intervals.”
