# Supplementary material for: Rubus coreanus extract prevents kidney fibrosis through TGF-β/Smad pathway inhibition
Source: PLoS One. 2025 May 12;20(5):e0321282. doi: 10.1371/journal.pone.0321282 (PMC12068887; doi:10.1371/journal.pone.0321282)
Supplement: Attachment — Submitted filename: Rebuttal letter_MN705_240221_231210_240630.docx [file pone.0321282.s005.docx]

**Comments from the Editor and Reviewers**

PONE-D-23-21717

Rubus coreanus extract attenuates kidney fibrosis through TGF-β/Smad pathway inhibition

PLOS ONE

**Editor comments:**

Dear Dr. Lee,

Thank you for submitting your manuscript to PLOS ONE. After careful consideration, we feel that it has merit but does not fully meet PLOS ONE’s publication criteria as it currently stands. Therefore, we invite you to submit a revised version of the manuscript that addresses the points raised during the review process.

Please submit your revised manuscript by Jun 02 2024 11:59PM. If you will need more time than this to complete your revisions, please reply to this message or contact the journal office at <a href="mailto:plosone@plos.org">plosone@plos.org. When you're ready to submit your revision, log on to https://www.editorialmanager.com/pone/ and select the 'Submissions Needing Revision' folder to locate your manuscript file.

Please include the following items when submitting your revised manuscript:

•	A rebuttal letter that responds to each point raised by the academic editor and reviewer(s). You should upload this letter as a separate file labeled 'Response to Reviewers'.

•	A marked-up copy of your manuscript that highlights changes made to the original version. You should upload this as a separate file labeled 'Revised Manuscript with Track Changes'.

•	An unmarked version of your revised paper without tracked changes. You should upload this as a separate file labeled 'Manuscript'.

If you would like to make changes to your financial disclosure, please include your updated statement in your cover letter. Guidelines for resubmitting your figure files are available below the reviewer comments at the end of this letter.

If applicable, we recommend that you deposit your laboratory protocols in protocols.io to enhance the reproducibility of your results. Protocols.io assigns your protocol its own identifier (DOI) so that it can be cited independently in the future. For instructions see: https://journals.plos.org/plosone/s/submission-guidelines#loc-laboratory-protocols. Additionally, PLOS ONE offers an option for publishing peer-reviewed Lab Protocol articles, which describe protocols hosted on protocols.io. Read more information on sharing protocols at https://plos.org/protocols?utm_medium=editorial-email&utm_source=authorletters&utm_campaign=protocols.

We look forward to receiving your revised manuscript.

Kind regards,

Qiang Ding, Ph.D.

Academic Editor

PLOS ONE

**Response:** The authors appreciate the decision and comments from the editor and reviewers. important comment. The authors have made the necessary improvements. In this revision, we have further addressed the implication and limitation of this study. Thank you again for the time and opportunity for this revision. Through the revision process, the authors were able to fill in the gaps and provide a more logical explanation for our findings.

The required files of a rebuttal letter (file labeled 'Response to Reviewers'), a marked-up copy of the manuscript (file labeled 'Revised Manuscript with Track Changes'), and an unmarked version of the revised paper without tracked changes ('Manuscript') were all prepared and uploaded. Financial disclosures are newly added to the editorial manager system. The authors have endeavored to follow the guidelines for resubmitting figure files. The authors ensure that this manuscript meets PLOS ONE's style requirements.

With this revision, we hope to have this study published in PLOS ONE. Thank you very much.

**Reviewers' comments:**

**Reviewer #2:**

There are some major weaknesses to be addressed. One is the model is preventative, don't support its role in treatment of kidney fibrosis, it should be evaluated after kidney fibrosis established. Otherwise, its best role is preventative, not treatment.

Even preventative, the data has major flaws. HK2 cells, not primary cells used for the tested. The drug kinetics and time- dose-effects are not evaluated, significantly reduced the confidence of the research.

**Response:** Thank you for the important comment. I agree with the reviewer's point. In this study, Rubus coreanus extract was administered before the development of fibrosis, and therefore, the preventive effect (not therapeutic effect) of Rubus coreanus extract was verified in this study. Therefore, we have revised the title of the manuscript from "Rubus coreanus extract attenuates kidney fibrosis through TGF-β/Smad pathway inhibition" to "Rubus coreanus extract prevents kidney fibrosis through TGF-β/Smad pathway inhibition".

In the abstract, “we aimed to investigate the efficacy of water-soluble extract of Rubus coreanus (MN705) in attenuating kidney fibrosis in a mouse model of unilateral ureteral obstruction (UUO) and in an *in vitro* model of TGF-β-challenged HK-2 cells.” > “we aimed to investigate the efficacy of water-soluble extract of Rubus coreanus (MN705) in preventing kidney fibrosis in a mouse model of unilateral ureteral obstruction (UUO) and in an *in vitro* model of TGF-β-challenged HK-2 cells.”; “a potential target for treatment of kidney fibrosis.” > “a potential target for prevention of kidney fibrosis.

In the discussion, we also stated that “In addition, this study only confirmed the preventive effect of Rubus coreanus on anti-fibrosis. Further experiments and clinical studies are necessary to determine if the Rubus coreanus has a therapeutic effect on kidney fibrosis.”

At this stage of revision, it is beyond the capabilities of my lab to replicate this experimental study with primary kidney tubular cells rather than HK2 cells. Therefore, I have included these limitations in the Discussion section as follows, “This study showed the effects of Rubus coreanus only in the UUO mice kidney fibrosis model and TGF-β challenged HK2 cellular model, which may be a limitation in generalizing the results of this study.”

In this study, the dose-dependent effect of MN705 (Rubus coreanus extract) was demonstrated in cellular and animal models, as shown in Fig. 2, Fig. 3 (*in vitro*, MN705 0.05, 0.1, 0.2 mg/ml), and Fig. 4, Fig. 5, Fig. 6 (*in vivo*, MN705 300, 600 mg/day). In cellular experiments, a dose-dependent improvement of fibrosis markers (fibronectin, collagen1) and an increase in antioxidants (MnSOD) were observed (Fig. 2). Animal studies also confirmed that alpha-SMA, collagen1 fibrosis markers decreased at high concentrations and pSmad 2/3 decreased in a concentration-dependent manner with increasing doses of MN705. 8-OHDG, a marker of oxidative damage, also decreased significantly with increasing MM705 concentration.

Although the study has some limitations, this is a meaningful study that demonstrates the protective effect of MN705 (Rubus coreanus extract) against kidney fibrosis in cellular and animal models. Especially, this study has advantages since the results were improved by conducting revisions as pointed out by the editors and reviewers. We hope that this revision will give this study the opportunity to be published in the Plos One.
