# Supplementary material for: Rubus coreanus extract prevents kidney fibrosis through TGF-β/Smad pathway inhibition
Source: PLoS One. 2025 May 12;20(5):e0321282. doi: 10.1371/journal.pone.0321282 (PMC12068887; doi:10.1371/journal.pone.0321282)
Supplement: Attachment — Submitted filename: Rebuttal letter_MN705_241031.docx [file pone.0321282.s006.docx]

**Comments from the Editor and Reviewers**

PONE-D-23-21717

Rubus coreanus extract prevents kidney fibrosis through TGF-β/Smad pathway inhibition

PLOS ONE

**Editor comments:**

Dear Dr. Lee,

Thank you for submitting your manuscript to PLOS ONE. After careful consideration, we feel that it has merit but does not fully meet PLOS ONE’s publication criteria as it currently stands. Therefore, we invite you to submit a revised version of the manuscript that addresses the points raised during the review process.

==============================

Reviewer is not satisfied with the response. Detailed comments included. Please address the reviewer's comments accordingly.

==============================

Please submit your revised manuscript by Sep 23 2024 11:59PM. If you will need more time than this to complete your revisions, please reply to this message or contact the journal office at <a href="mailto:plosone@plos.org">plosone@plos.org. When you're ready to submit your revision, log on to https://www.editorialmanager.com/pone/ and select the 'Submissions Needing Revision' folder to locate your manuscript file.

Please include the following items when submitting your revised manuscript:

•	A rebuttal letter that responds to each point raised by the academic editor and reviewer(s). You should upload this letter as a separate file labeled 'Response to Reviewers'.

•	A marked-up copy of your manuscript that highlights changes made to the original version. You should upload this as a separate file labeled 'Revised Manuscript with Track Changes'.

•	An unmarked version of your revised paper without tracked changes. You should upload this as a separate file labeled 'Manuscript'.

If you would like to make changes to your financial disclosure, please include your updated statement in your cover letter. Guidelines for resubmitting your figure files are available below the reviewer comments at the end of this letter.

If applicable, we recommend that you deposit your laboratory protocols in protocols.io to enhance the reproducibility of your results. Protocols.io assigns your protocol its own identifier (DOI) so that it can be cited independently in the future. For instructions see: https://journals.plos.org/plosone/s/submission-guidelines#loc-laboratory-protocols. Additionally, PLOS ONE offers an option for publishing peer-reviewed Lab Protocol articles, which describe protocols hosted on protocols.io. Read more information on sharing protocols at https://plos.org/protocols?utm_medium=editorial-email&utm_source=authorletters&utm_campaign=protocols.

We look forward to receiving your revised manuscript.

Kind regards,

Qiang Ding, Ph.D.

Academic Editor

PLOS ONE

**Response:** The authors appreciate the decision and comments from the editor and reviewers’ important comments. The authors have made the necessary improvements. In this revision, we performed *in vitro* tests with human primary renal proximal tubule epithelial cells and validated the findings from the HK-2 cell line presented in the original manuscript. In addition, we have further addressed the implication and limitation of this study. Thank you again for the time and opportunity for this revision. Through the revision process, the authors were able to fill in the gaps and provide a more logical explanation for our findings.

The required files of a rebuttal letter (file labeled 'Response to Reviewers'), a marked-up copy of the manuscript (file labeled 'Revised Manuscript with Track Changes'), and an unmarked version of the revised paper without tracked changes ('Manuscript') were all prepared and uploaded. Financial disclosures are included in the editorial manager system. The authors have endeavored to follow the guidelines for resubmitting figure files. The authors ensure that this manuscript meets PLOS ONE's style requirements.

With this revision, we hope to have this study published in PLOS ONE. Thank you very much.

**Reviewers' comments:**

**Reviewer #2:**

Congratulations for author’s arguments that there are difficulties in addressing the concerns. However, here are minimal requirements in order to meet with review criteria.

1. The limitation added to the discussion is very short and too brief, and should be enough for readers to understand the limitation of the study. It needs to include specifics such as that this work is not done in primary cells and there are limitations for this.

**Response:** Thank you for the important comment. I agree with the reviewer's point. In response to the reviewer's constructive suggestions for enhancing this research, we performed additional *in vitro* tests using human renal proximal tubular epithelial cells (RPTEC, Lonza, Catalog #: CC-2553).

In an *in vitro* test using RPTEC, we were able to confirm the results obtained with HK-2 cells. The antioxidant MnSOD increased after MN705 treatment, while fibrosis-associated Smad2/3, phospho-smad2/3, and fibronectin decreased. The results are incorporated as Figure 4 and detailed as follows: “The results found in the HK-2 cell line were validated using human renal proximal tubular epithelial cells. After MN705 treatment, the epithelial cell morphology altered by TGF-β 2 ng/ml for 48-h was restored to a more elongated and spindle-shaped morphology (Fig. 4A). Western blots were employed to investigate alterations of proteins associated with anti-oxidation and kidney fibrosis. MN705 led to enhanced expression of the antioxidant MnSOD, reduced fibronectin levels, and diminished phospho-Smad2/3 (Fig. 4B, C).”

In addition, we describe the implications and limitations of our study in more detail as follows: “This study has the unique advantage of being the first to show the antifibrotic properties of *Rubus coreanus* in kidney fibrosis. Additionally, it proposes that the modulation of TGF-β/Smad signaling, along with the anti-oxidative effects, is the underlying mechanism of anti-fibrosis. In addition, the effects of *Rubus coreanus* on TGF-β/Smad signaling, antioxidants, and preventing fibrosis were confirmed in both animal study and two *in vitro* models. While these studies suggest a potential therapeutic role for antioxidants in kidney fibrosis, it's essential to approach the findings with caution. The effectiveness of antioxidants may vary depending on the specific conditions and causes of kidney fibrosis. The UUO model induces kidney fibrosis through a sustained pressure effect within the kidney. This pathophysiological trait contrasts with most clinical disorders associated with kidney fibrosis. In addition, TGF-β is a principal pro-fibrotic factor that activates myofibroblasts and serves as a crucial mediator in the progression of kidney fibrosis. However, it is essential to experimentally replicate many circumstances associated with kidney fibrosis, including hypoxia, infection, and inflammation, to facilitate the generalization of experimental results. In addition, this study only confirmed the preventive effect of Rubus coreanus on anti-fibrosis. Further experiments and clinical studies are necessary to determine if the Rubus coreanus has a therapeutic effect on kidney fibrosis. At last, the anti-fibrotic impact of Rubus coreanus was only observed in the 7-day model of the UUO mouse. Therefore, it is worthwhile to investigate if this anti-fibrotic effect persists over shorter or longer time intervals.”

2. This reviewer understands that authors may not be able to replicate all or most of the data in primary cells at this stage, but, it is necessary to replicate or confirm some key findings, even one or two results, to satisfy the minimal requirement. This is minimal requirement as this stage. Otherwise, the scientific value is totally not convinced.

Response: Thank you for the critical and valuable comment. I totally agree with the reviewer's point. Previously, we utilized HK-2 cells, a representative renal tubule cell line, to demonstrate the mechanism of the anti-fibrotic effect of Rubus coreanus in the kidney, focusing on the time-dependent alterations in the Smad pathway during TGF-β-induced fibrosis. Nonetheless, owing to the disparities between animal and human kidney cells, one may doubt the applicability of results obtained from animal cells to human. In response to the reviewer's constructive suggestions for enhancing this research, we procured human renal proximal tubular epithelial cells (RPTEC, Lonza, Catalog #: CC-2553) and performed additional *in vitro* studies.

In an *in vitro* test using RPTEC, we were able to confirm the results obtained with HK-2 cells. The antioxidant MnSOD increased after MN705 treatment, while fibrosis-associated Smad2/3, phospho-smad2/3, and fibronectin decreased. The results are incorporated as Figure 4 and detailed as follows: “The results found in the HK-2 cell line were validated using human renal proximal tubular epithelial cells. After MN705 treatment, the epithelial cell morphology altered by TGF-β 2 ng/ml for 48-h was restored to a more elongated and spindle-shaped morphology (Fig. 4A). Western blots were employed to investigate alterations of proteins associated with anti-oxidation and kidney fibrosis. MN705 led to enhanced expression of the antioxidant MnSOD, reduced fibronectin levels, and diminished phospho-Smad2/3 (Fig. 4B, C).”

We thank the editors and reviewers for their important comments during the three revisions, which improved the paper. We hope that this revision will give this study the opportunity to be published in the Plos One.
