# Supplementary material for: Rubus coreanus extract prevents kidney fibrosis through TGF-β/Smad pathway inhibition
Source: PLoS One. 2025 May 12;20(5):e0321282. doi: 10.1371/journal.pone.0321282 (PMC12068887; doi:10.1371/journal.pone.0321282)
Supplement: Attachment — Submitted filename: Rebuttal letter_MN705_250107.docx [file pone.0321282.s007.docx]

**Comments from the Editor and Reviewers**

PONE-D-23-21717

Rubus coreanus extract prevents kidney fibrosis through TGF-β/Smad pathway inhibition

PLOS ONE

**Editor comments:**

Dear Dr. Lee,

Thank you for submitting your manuscript to PLOS ONE. After careful consideration, we feel that it has merit but does not fully meet PLOS ONE’s publication criteria as it currently stands. Therefore, we invite you to submit a revised version of the manuscript that addresses the points raised during the review process.

==============================

Reviewer raised serious question about the conflict interest for your revision, in addition to the scientific data supporting the conclusion, and data interpretation and if sufficient consideration has been given to the potential confounding factors that may affect the supporting data and conclusion.

==============================

Please submit your revised manuscript by Feb 10 2025 11:59PM. If you will need more time than this to complete your revisions, please reply to this message or contact the journal office at plosone@plos.org. When you're ready to submit your revision, log on to https://www.editorialmanager.com/pone/ and select the 'Submissions Needing Revision' folder to locate your manuscript file.

Please include the following items when submitting your revised manuscript:

•	A rebuttal letter that responds to each point raised by the academic editor and reviewer(s). You should upload this letter as a separate file labeled 'Response to Reviewers'.

•	A marked-up copy of your manuscript that highlights changes made to the original version. You should upload this as a separate file labeled 'Revised Manuscript with Track Changes'.

•	An unmarked version of your revised paper without tracked changes. You should upload this as a separate file labeled 'Manuscript'.

If you would like to make changes to your financial disclosure, please include your updated statement in your cover letter. Guidelines for resubmitting your figure files are available below the reviewer comments at the end of this letter.

If applicable, we recommend that you deposit your laboratory protocols in protocols.io to enhance the reproducibility of your results. Protocols.io assigns your protocol its own identifier (DOI) so that it can be cited independently in the future. For instructions see: https://journals.plos.org/plosone/s/submission-guidelines#loc-laboratory-protocols. Additionally, PLOS ONE offers an option for publishing peer-reviewed Lab Protocol articles, which describe protocols hosted on protocols.io. Read more information on sharing protocols at https://plos.org/protocols?utm_medium=editorial-email&utm_source=authorletters&utm_campaign=protocols.

We look forward to receiving your revised manuscript.

Kind regards,

Qiang Ding, Ph.D.

Academic Editor

PLOS ONE

**Response:** The authors appreciate the decision and comments from the editor and reviewers’ important comments. During the three rounds of revisions, we diligently addressed the editor's and reviewers' requests by supplementing the experimental content and preparing thorough responses. During the third revision process, an investigator from the company that provided the natural product conducted the proofreading of the manuscript. This individual had initially provided the natural product and participated in the early stages of the research purely for academic purposes. Furthermore, she also contributed to the cell experiments added during the revision process. Based on these contributions and with the consent of the other authors, we deemed the investigator qualified for authorship and added she as a co-author.

However, the reviewer pointed out that adding an author with a potential conflict of interest during the revision process could raise significant ethical concerns. All authors agree that including an investigator from the material-supplying company as a co-author and explicitly indicating their affiliation with the company may conflict with ethical standards regarding conflicts of interest. Therefore, to demonstrate that the natural product was provided solely for academic purposes and to ensure there are no conflicts of interest, we have decided to exclude the company and its representative from the list of authors with consents of all authors. We kindly ask for your understanding regarding the additional critique raised by the reviewer, as it pertains to new research directions that are difficult to pursue at this stage of the study.

Thank you again for the time and opportunity for this revision. Through the revision process, the authors were able to fill in the gaps and provide a more logical explanation for our findings. The required files of a rebuttal letter (file labeled 'Response to Reviewers'), a marked-up copy of the manuscript (file labeled 'Revised Manuscript with Track Changes'), and an unmarked version of the revised paper without tracked changes ('Manuscript') were all prepared and uploaded. Financial disclosures are included in the editorial manager system. The authors have endeavored to follow the guidelines for resubmitting figure files. The authors ensure that this manuscript meets PLOS ONE's style requirements.

With this revision, we hope to have this study published in PLOS ONE. Thank you very much.

**Reviewers' comments:**

**Reviewer #2:**

The authors have declared that no competing interests exist. However, this revision has serous conflict interest to be addressed.

This revision has included the company, Medvill Co., Ltd., which provides the testing drug/ Rubus coreanus extract extract powders used for the study, and this company was not included in any of the prior versions. This is a serious issue. One is that providing material is not warranty an authorship, based on the journal guideline. Authors’ contribution is vague and no specifics for why the company is added to the paper. Second, most importantly, conflict interest. The data are now considered directly related to the company benefit and the results and adding the company as the authorship promote the company product and image by this manuscript. Therefore, these are potential conflict interest, and the results have to be re-evaluated as a promoting manuscript for the company’s product, rather than only a purely scientific research paper with insufficient data to support the conclusion.

Giving the nature of this manuscript is therapeutic intervention, this could serve as an advertisement for the company, a serious conflict interest, as the company is added at the last minute, and this version and last version only have minimal difference between them.

As these changes, this reviewer would like to required more in vivo evidence for the prevention. Current results only indicates reduction of some pathological changes.

Mostly importantly, the figures and labels are low quality, unclear, difficult to judge.

Limited animal data to support the conclusion. Figs 5-7 are from the animal studies, figures are low quality and unclear. The connection between the figures are not studied.

Major weaknesses as following:

1. There is no evaluation of pharmacologic toxicity in animals; 2. the internal drug amount such as concentration in blood and kidney tissues.; 3. What are the mechanism for the MN705 reduces TGFbeta signaling and matrix deposition? 4. Is the drug MN705 binds indirectly or directly to TGFbeta receptor, or what are the action route?; 5. Why NN705, is it better than using anti-TGFbeta inhibitors directly?

6. Inhibition of TGFbeta may raise cancer concern. How about MN705, any concern, if only targeting TGFbeta?; 7. is MN705 permeable to cells and kidney tissues?; 8. How the MN705 affects MnSOD?

9. And how MnSOD is related to TGFbeta signaling, and UUO kidney fibrosis?; 10. Is MnSOD increase by MN705 a result or critical pathway?; 11. What if MnSOD is blocked, and animal are treated with MN705, will kidney fibrosis changes during these conditions?

**Response:** Thank you for the important comment. I agree with the reviewer's point. During the three rounds of revisions, we diligently addressed the editor's and reviewers' requests by supplementing the experimental content and preparing thorough responses. During the third revision process, an investigator from the company that provided the natural product conducted the proofreading of the manuscript. This individual had initially provided the natural product and participated in the early stages of the research purely for academic purposes. Furthermore, they contributed to the cell experiments added during the revision process. Based on these contributions and with the consent of the other authors, we deemed the investigator qualified for authorship and added she as a co-author.

However, the reviewer pointed out that adding an author with a potential conflict of interest during the revision process could raise significant ethical concerns. All authors agree that including an investigator from the material-supplying company as an author and explicitly indicating their affiliation with the company may conflict with ethical standards regarding conflicts of interest. Therefore, to demonstrate that the natural product was provided solely for academic purposes and to ensure there are no conflicts of interest, we have decided to exclude the company and its representative from the list of authors. We kindly ask for your understanding regarding the additional critique raised by the reviewer, as it pertains to new research directions that are difficult to pursue at this stage of the study.

In addition, the reviewer also pointed out that the quality of the presented images is not satisfactory. Upon reviewing the images included in the PDF, we agree that the resolution appears low. However, when viewing the original images we provided for download, the high-resolution versions are clearly visible. If necessary, we can submit individual files for each image separately.

**Response to Q1**. This study primarily aims to investigate the effects of *Rubus coreanus* extract on kidney disease. In the cellular model, toxicity was assessed using the MTT assay. While there is a potential risk of toxicity at higher concentrations in *in vivo* experiments, no toxicity was observed within the design of this study. Although the possibility of toxicity at high concentrations exists, this study holds value as a report focused on verifying the efficacy of *Rubus coreanus* extract.

**Response to Q2**. *Rubus coreanus* extract is a natural compound complex composed of various components, making it challenging to measure its concentration in blood and kidney tissues.

**Response to Q3**. Thank you for important comments. As presented in the results of this study, MN705 appears to alleviate fibrosis by regulating the TGF-β/Smad pathway and mitigating oxidative damage. Further studies are needed to elucidate the underlying mechanisms by which MN705 regulates the TGF-β/Smad pathway and reduces oxidative damage.

**Response to Q4**. Thank you for important comments. *Rubus coreanus* extract is a natural compound complex composed of various components. Therefore, further studies are needed to identify which specific components bind to the TGF-β receptor and to elucidate the mechanisms by which they mediate signaling.

**Response to Q5**. In this study, we validated the anti-fibrotic effects of *Rubus coreanus* extract in a kidney disease model but did not compare its effects with other TGF-β inhibitors. Comparative studies with other TGF-β inhibitors should be conducted in future research.

**Response to Q6**. As previously explained, *Rubus coreanus* extract is a natural compound complex composed of various components. It is thought to alleviate fibrosis in kidney disease models through multiple mechanisms. In this study, we focused on elucidating its mechanisms, particularly in regulating TGF-β signaling and mitigating oxidative damage. Many therapeutic agents currently used for chronic diseases provide significant benefits in alleviating disease but may also have associated side effects. As a natural compound complex, *Rubus coreanus* extract is noteworthy for its potential to offer beneficial biological effects with relatively fewer side effects. Beyond the TGF-β signaling and oxidative damage mitigation mechanisms identified in this study, other potential mechanisms warrant further investigation in future research.

**Response to Q7**. As previously explained, *Rubus coreanus* extract is a natural compound complex composed of various components. It is thought to alleviate fibrosis in kidney disease models through multiple mechanisms. In this study, we focused on elucidating its mechanisms, particularly in regulating TGF-β signaling and mitigating oxidative damage. Many therapeutic agents currently used for chronic diseases provide significant benefits in alleviating disease but may also have associated side effects. As a natural compound complex, *Rubus coreanus* extract is noteworthy for its potential to offer beneficial biological effects with relatively fewer side effects. Beyond the TGF-β signaling and oxidative damage mitigation mechanisms identified in this study, other potential mechanisms warrant further investigation in future research.

**Response to Q8**. Thank you for important comments. As presented in the results of this study, MN705 appears to alleviate fibrosis by regulating the TGF-β/Smad pathway and mitigating oxidative damage. Further studies are needed to elucidate the underlying mechanisms by which MN705 reduces oxidative damage via MnSOD.

**Response to Q9**. This study aimed to investigate the anti-fibrotic effects of MN705 in a kidney disease model, and as such, the mechanisms by which MnSOD influences TGF-β signaling and fibrosis fall outside the scope of this research. However, relevant insights can be drawn from prior studies. Increased oxidative stress can activate TGF-β signaling, leading to fibrosis and tissue remodeling in various diseases. Importantly, high MnSOD activity can counteract oxidative stress, thereby inhibiting excessive TGF-β activation and potentially preventing fibrosis development (J Gerontol A Biol Sci Med Sci. 2015;70(5):533. doi: 10.1093/gerona/glu090). Additionally, studies have demonstrated that increased MnSOD expression or activity can suppress TGF-β signaling by reducing oxidative stress, potentially limiting fibrosis development in various tissues (Int J Mol Sci. 2022;23(24):15893. doi: 10.3390/ijms232415893).

**Response to Q10** **and Q11.** Based on the results of this study, it is inferred that MN705 is directly involved in the upregulation of MnSOD. The anti-fibrotic effects of MN705 may be attenuated when MnSOD is inhibited. However, as MN705 is a natural extract composed of various components, there is also a possibility that its anti-fibrotic effects are mediated through other mechanisms. Further studies are required to determine which specific components of Rubus coreanus extract contribute to its anti-fibrotic effects in kidney disease and through which mechanisms these effects are achieved.

12. Why Fig.6A missing one lane of proteins for “UUO+MN705 600mg” condition? Is the experiment only done once?

**Response to Q12:** Thanks for your comment. As described in the Methods, male C57BL/6 mice (7 weeks old) were randomly assigned to the following groups: sham/vehicle (distilled water, n = 4), sham/MN705 (600 mg/kg/day, n = 4), UUO/vehicle (n = 6), UUO/MN705-low dose (300 mg/kg/day, n = 6), and UUO/MN705-high dose (600 mg/kg/day, n = 6). Western blot analysis was conducted in two separate sets, with the samples divided as follows: sham/vehicle (2 lanes), sham/MN705 (2 lanes), UUO/vehicle (3 lanes), UUO/MN705-low dose (3 lanes), and UUO/MN705-high dose (3 lanes). Our Figure 6A UUO+MN705 600mg did not lack lanes (3 lanes). And we had already attached the western bands that were only used for statistical purposes in the Supplements (western band raw, PAGE 15). All results presented in this study were validated through 2–3 repeated experiments.

13. Also, Fig.6 does not show much diffierence for pSmad2/3 levels between UUO and UUO+MN705, but the fibrosis is difference. Any explanation?

**Response to Q13:** Thank you for important comment. The reviewer pointed out that the changes in pSmad presented in Fig. 6A do not appear significant. As previously explained, the western blot analysis was conducted in two separate sets, and statistical significance was confirmed when all the results were combined and analyzed. Please also review the additional western blot results provided in the supplementary materials.

Supplements (western band raw, PAGE 15).

We thank the editors and reviewers for their important comments during the three revisions, which improved the paper. We hope that this revision will give this study the opportunity to be published in the Plos One.
